# S100A8/A9‐High Macrophages Activate Intestinal Fibroblasts via mCCL6/hCCL15‐CCR1 Axis to Drive Intestinal Fibrosis in Crohn's Disease

**DOI:** 10.1002/advs.76353

**Published:** 2026-06-29

**Authors:** Shu Wang, Jiayun Wang, Junjie Lin, Ziping Ye, Geyujia Zhou, Jingjing Ma, Junjian Sun, Jiang Yu, Yingdi Zhang, Nana Tang, Chunhua Jiao, Xiaojing Zhao, Hongjie Zhang

**Affiliations:** ^1^ Department of Gastroenterology The First Affiliated Hospital with Nanjing Medical University Nanjing Jiangsu China

**Keywords:** Crohn's disease, intestinal fibrosis, mCCL6/ hCCL15, S100A8/A9‐high macrophages

## Abstract

Intestinal fibrosis presents a major clinical challenge in Crohn's disease (CD) due to the lack of effective pharmacological interventions. The underlying mechanisms of intestinal fibrosis remain largely elusive. Reanalysis of the single‐cell RNA‐seq data from full‐thickness CD tissue identifies a distinct profibrotic macrophage subset characterized by high *S100A8* and *S100A9* expression. CellChat analysis indicates strong communication between this S100A8/A9‐high (S100A8/A9^hi^) macrophage subset and fibroblasts. Adoptive transfer of S100A8/A9^hi^ macrophages exacerbate intestinal fibrosis in mice with chronic dextran sulfate sodium (DSS)‐induced colitis. Consequently, pharmacological inhibition of S100A8/A9 significantly ameliorates intestinal fibrosis in murine chronic colitis. Proteomic analysis further identifies murine CCL6 (mCCL6) as the key pro‐fibrotic mediator secreted by S100A8/A9^hi^ macrophages, which acts via CC chemokine receptor 1 (CCR1) to regulate fibroblasts. Antibody blockade of mCCL6 alleviates established intestinal fibrosis in a DSS‐induced colitis model. Mechanistically, S100A8/A9^hi^ macrophages drive mCCL6 production via STAT3 activation. Similarly, the human ortholog of CCL6, CCL15 (hCCL15), exerts pro‐fibrotic effects on fibroblasts via the CCR1 receptor. Our findings reveal that targeting S100A8/A9^hi^ macrophage may be a therapeutic strategy against intestinal fibrosis in CD.

## Graphical abstract

S100A8/A9‐high macrophages are markedly enriched in the stenotic intestinal tissue of patients with Crohn's disease. These profibrotic macrophages secrete mCCL6 in a STAT3‐dependent manner. mCCL6 and its human ortholog hCCL15 activate fibroblasts via the CCR1 receptor, thereby driving excessive collagen deposition. Pharmacological targeting of this S100A8/A9–mCCL6/hCCL15–CCR1 signaling axis effectively suppresses intestinal fibrosis development and progression.

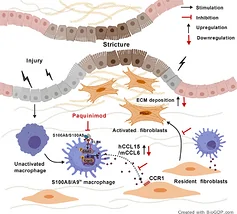

## 1 Introduction

Crohn's disease (CD) is a progressive disorder pathologically characterized by transmural inflammation that instigates fibrotic remodeling, resulting in fibrous tissue deposition that obliterates the normal parenchyma and leads to progressive luminal narrowing [1]. As no approved anti‐fibrotic therapies are currently available [2], approximately 75% of patients with CD‐associated fibrosis ultimately require surgical intervention [3, 4]. Intestinal fibrosis results from a dysregulated wound healing response, defined by the excessive accumulation of extracellular matrix (ECM) components [1, 5, 6], which is driven primarily by activated fibroblasts upon inflammatory or injurious stimuli [7]. While progress has been made in characterizing CD‐associated fibrosis, the specific molecular drivers of fibroblast activation and fibrotic progression have yet to be fully elucidated.

Beyond the effector functions of fibroblasts themselves, the immune microenvironment—particularly the regulatory networks governing tissue repair—is critically involved in initiating and sustaining the fibrotic cascade [8]. Macrophages are immune sentinels that orchestrate tissue homeostasis [9] and are defined by their heterogeneity and remarkable phenotypic plasticity [10, 11], which enables them to adopt a spectrum of functionally distinct states shaped by the local microenvironment [12]. Given their considerable plasticity and heterogeneity, macrophages function as central drivers of fibrogenesis during aberrant tissue repair [13, 14]. Macrophages functionally shift from reparative sentinels in homeostasis to pro‐fibrotic drivers upon dysregulated activation [14, 15]. For example, macrophages have been shown to assemble a vitronectin‐enriched extracellular niche to aggravate kidney fibrosis [16]; conversely, their depletion suppressed renal fibrosis progression in a repeated low‐dose cisplatin model [17] and limited fibrotic injury in the onset of liver fibrosis [18]. Despite the well‐established profibrotic roles of macrophages in various organ fibroses, their roles in CD‐associated intestinal fibrosis remain largely unexplored.

In this study, we first identified a distinct S100A8/A9‐high (S100A8/A9^hi^) macrophage subset by reanalyzing scRNA‐seq data from full‐thick CD intestinal tissues and confirmed their significant infiltration into stenotic regions. To determine their causal role in fibrosis, we employed adoptive transfer of bone marrow‐derived S100A8/A9^hi^ macrophages in a mouse model of dextran sulfate sodium (DSS)‐induced colitis. Having established their profibrotic capacity in vivo, we investigated the therapeutic effects of targeting S100A8/A9 in chronic colitis. To identify underlying mediators, we performed proteomic profiling and established a co‐culture system. Mechanistically, we elucidated how S100A8/A9^hi^ macrophages regulate mouse CCL6 (mCCL6) production. Finally, we demonstrated that the human ortholog of mCCL6, CCL15 (hCCL15), exerts CC chemokine receptor 1 (CCR1)‐dependent profibrotic effects on the proliferation, activation, and ECM production of fibroblasts.

## 2 Results

### 2.1 S100A8/A9^hi^ Macrophages Exhibit Increased Infiltration in Stenotic CD

To investigate immune cell dynamics in intestinal fibrosis, we analyzed a scRNA‐seq dataset of full‐thickness intestinal specimens from CD patients, encompassing non‐involved, inflamed (non‐stricture), and stricture regions. Building on prior reports of enhanced fibroblast‐myeloid interactions in strictures [19], we focused on the myeloid compartment. Unsupervised clustering of myeloid cells revealed 15 distinct subpopulations (Figure 1A and Figure S1A,B). We then employed the computational tool Single Cell Distance (scDist) [20] to quantify intercellular distances and compare these subpopulations across the three patient groups. Notably, S100A8/A9^hi^ macrophage exhibited the greatest heterogeneity, ranking highest in cellular distance scores in both inflamed versus non‐involved and stricture versus inflamed comparisons (Figure 1B). This finding highlights the heterogeneity of S100A8/A9^hi^ macrophages, positioning them as a potential key driver of fibrosis in CD. Consistently, the proportion of S100A8/A9^hi^ macrophages was significantly elevated in stenotic sites (Figure 1C). CellChat analysis of stricture tissue further revealed robust interaction networks between the S100A8/A9^hi^ macrophage subset and fibroblasts (Figure 1D), supporting their central role in fibrotic crosstalk.

**FIGURE 1:**
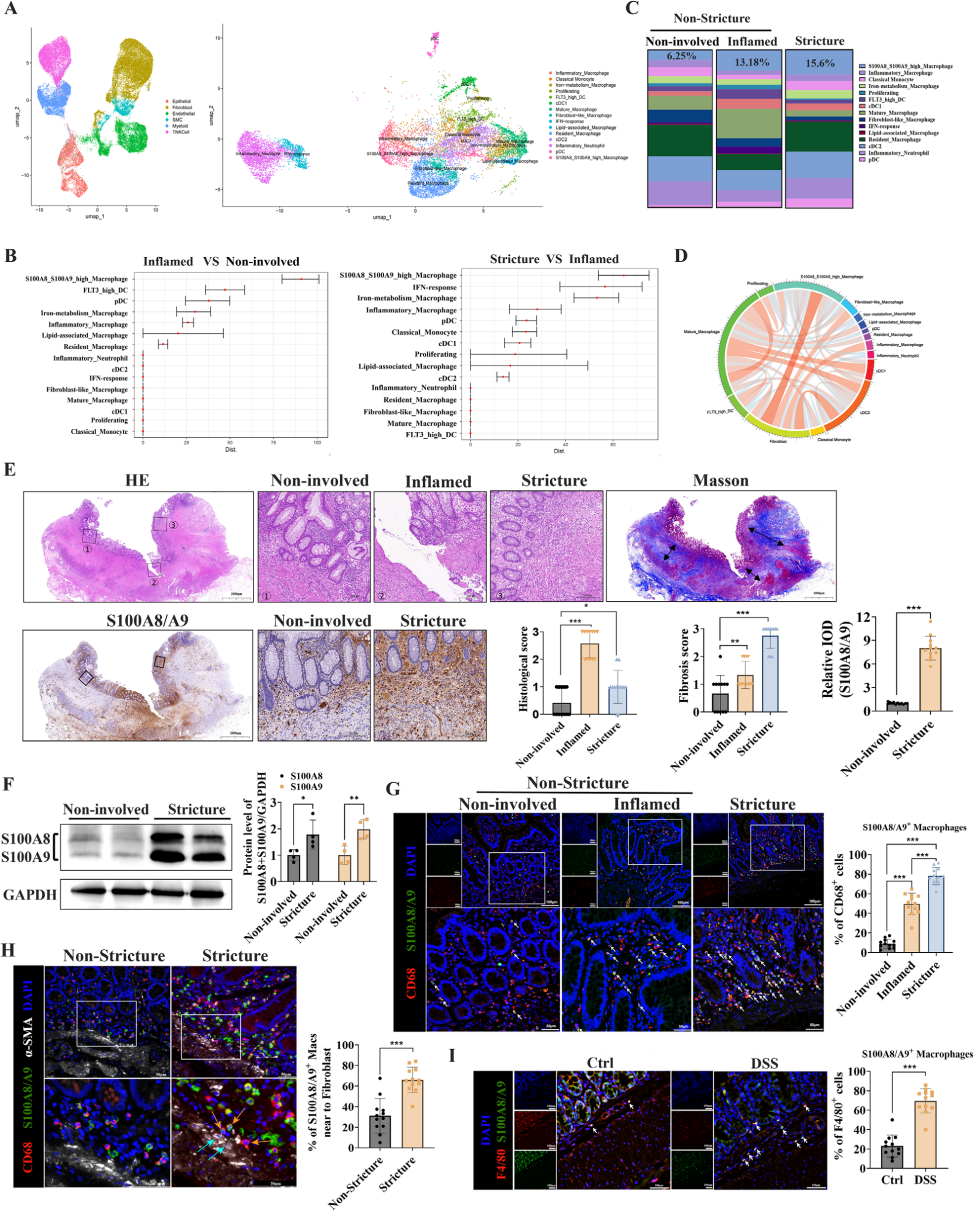
S100A8/A9^hi^ macrophages abundantly infiltrate fibrotic intestinal regions of Crohn's disease (CD). (A) Uniform manifold approximation and projection (UMAP) visualization of the single‐cell atlas and compartmental UMAP plots for myeloid cells. (B) Cellular distribution distances of myeloid cells between inflamed vs. non‐involved and stricture vs. inflamed were calculated using Single Cell Distance (ScDist) computational tool. (C) Proportion of the myeloid cell subsets in non‐involved, inflamed, and stricture regions. (D) Ligand‐receptor interactions were predicted by CellChat among myeloid cell subsets in stricture regions and fibroblasts. Lines connect receptor‐expressing cells (color‐indicated) to their cognate ligand‐expressing partners, with line thickness weighted by the number of potential ligand‐receptor pairs. (E) Representative images of Hematoxylin & eosin (H&E), Masson's trichrome, and S100A8/A9 immunohistochemical (IHC) staining of full‐thickness surgical sections from CD patients (non‐involved, inflamed, and stricture regions). (F) Protein levels of S100A8/A9 in CD stricture and non‐involved regions were measured by Western blot. (G) Representative immunofluorescence images and quantification of S100A8/A9^+^ macrophages (S100A8/A9^+^ CD68^+^) in the non‐involved, inflamed, and stricture intestine regions of CD patients. Scale bar: 100 µm (low magnification) and 50 µm (high magnification). *n* = 4 patients (3 fields per zone). (H) Immunofluorescence co‐localization analysis for S100A8/A9^+^ macrophages and fibroblasts in non‐stricture and stricture intestinal sections of CD. The proportion of S100A8/A9^+^ macrophages located within 30 µm of the nearest fibroblast in non‐stricture and stricture segments were calculated. Scale bar: 50 µm, *n* = 4 patients (3 fields per zone). (I) Immunofluorescence image and quantification for F4/80^+^ and S100A8/A9^+^ macrophages in colons from control (Ctrl) and chronic dextran sulfate sodium (DSS) colitis mice. Scale bar: 100 µm, *n* = 4 mice (3 fields per zone). Data are presented as means ± SD. ns, no significant difference, ^*^ *p* < 0.05, ^**^ *p* < 0.01, ^***^ *p* < 0.001.

We next performed histopathological validation. Hematoxylin and eosin (H&E) and Masson's trichrome staining of CD intestinal segments confirmed substantial submucosal expansion and collagen deposition in stenotic areas. Both immunohistochemical staining and Western blot analysis demonstrated higher levels of S100A8/A9 expression in stricture regions compared to non‐involved areas (Figure 1E, F). Further immunofluorescence analysis not only confirmed the enriched infiltration of S100A8/A9^+^ macrophage in stenotic CD (Figure 1G) but also revealed their close spatial interaction with activated fibroblasts at fibrotic foci (Figure 1H). A similarly marked infiltration was also observed in murine chronic colitic colons (Figure 1I), underscoring this as a consistent feature of the fibrotic mucosa. Collectively, these findings identify S100A8/A9^hi^ macrophages as a pivotal immune subset in the pathogenesis of intestinal fibrosis and a focus of future mechanistic investigation.

### 2.2 S100A8/A9^hi^ Macrophage Aggravates Intestinal Fibrosis in Chronic DSS‐Induced Colitis Mice

To explore the role of S100A8/A9^hi^ macrophages in intestinal fibrosis, we established an adoptive transfer model in DSS‐induced colitis mice. S100A8/A9^hi^ macrophages were generated in vitro by stimulating wild‐type (WT) bone marrow‐derived macrophages (BMDMs) with key inflammatory mediators associated with CD pathogenesis: lipopolysaccharide (LPS), interleukin‐1β (IL‐1β), and tumor necrosis factor‐α (TNF‐α). Among these, LPS emerged as the most potent and persistent inducer of both *S100a8* and *S100a9* expression, as validated by flow cytometry (Figure S2A,B). Chronic colitis was established in WT mice using a three‐cycle 2% DSS regimen. On day 5, clodronate liposomes were administered to deplete endogenous macrophages, with the depletion efficiency confirmed by flow cytometric analysis of splenocytes (Figure S2C). Beginning on day 7 post‐colitis induction, mice received weekly intravenous transfers of PBS, normal control BMDMs (NC‐BMDMs), or S100A8/A9^hi^ BMDMs, all pre‐labeled with the fluorescent dye DiR (Figure 2A). In vivo imaging confirmed the successful trafficking and colonization of transferred BMDMs within the lower abdomen and colon of recipient mice (Figure 2B).

**FIGURE 2:**
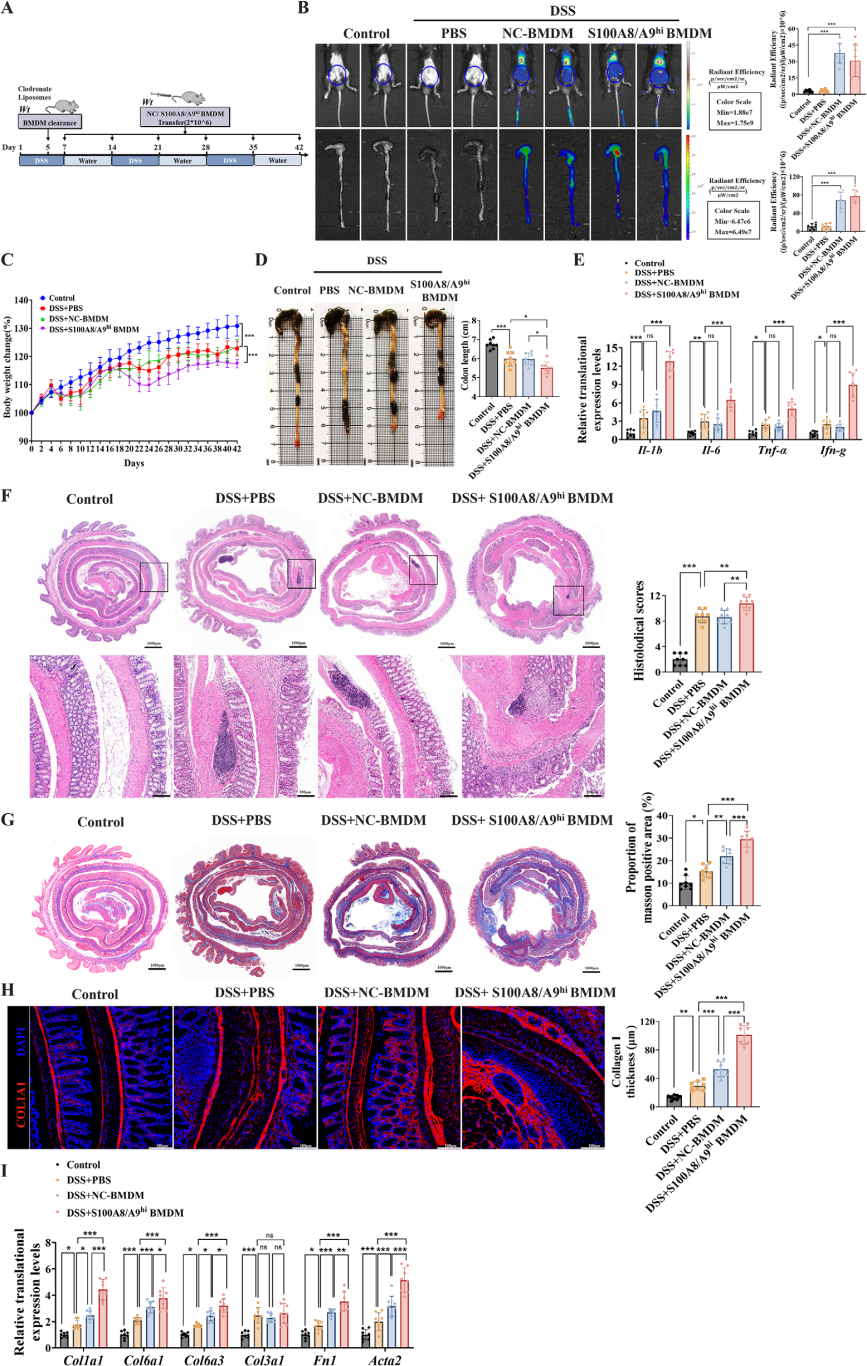
Adoptive transfer of S100A8/A9^hi^ macrophage exacerbates intestinal fibrosis in mice with DSS‐induced chronic colitis. C57BL/6J mice were subjected to three cycles of 2% DSS to induce chronic colitis. Bone marrow‐derived macrophages (BMDMs) were stimulated with LPS (100 ng/mL) for 24 h to generate an S100A8/A9 ‐high phenotype. Macrophages were depleted by tail vein injection of clodronate liposomes (200 µL/mouse) 48 h before BMDMs adoptive transfer. Mice were divided into four groups (*n* = 8/group) and treated on day 7 as follows: (1) Control group: received normal water throughout; (2) DSS+PBS group: received 200 µL PBS; (3) DSS+NC‐BMDM group: received injections of 2×10^6^ normal BMDMs (NC‐BMDM); (4) DSS+S100A8/A9^hi^ group: received injections of 2×10^6^ LPS‐induced S100A8/A9^hi^ BMDMs. All injections were administered via the tail vein weekly. (A) Experimental schematic of the adoptive transfer protocol in the DSS‐induced chronic colitis model. (B) Representative in vivo images of DiR‐labeled macrophages 24 h post‐injection and corresponding ex vivo fluorescence signals in colon tissues. (C) Body weight changes monitored every two days, presented as the percentage of initial body weight. (D) Representative images of colons and statistical analysis of colon length. (E) Transcriptional levels of inflammatory cytokines (*Il‐1b, Il‐6*, *Tnf‐α*, and *Ifn‐g*) in colonic tissues determined by RT‐qPCR. (F) Representative H&E staining and histological scores of colonic tissues. Scale bar: 1000 µm (low magnification) and 100 µm (high magnification). (G) Masson's trichrome staining and quantification of collagen deposition in colons. Scale bar: 1000 µm. (H) Immunofluorescence staining and quantification of collagen I‐positive layer thickness. Scale bar: 100 µm. (I) RT‐qPCR analysis of fibrosis‐associated genes (*Col1a1*, *Col6a1*, *Col6a3*, *Col3a1*, *Fn1*, and *Acta2*) in colonic tissues. All values are expressed as mean ± SD. ns, no significant difference, ^*^ *p* < 0.05, ^**^ *p* < 0.01, ^***^ *p* < 0.001.

The adoptive transfer of S100A8/A9^hi^ BMDMs significantly exacerbated colitis severity compared to that of NC‐BMDMs. This was evidenced by exacerbated body weight loss (Figure 2C), shortened colon length (Figure 2D), elevated levels of inflammatory cytokines (*Il‐1b*, *Il‐6, Tnf‐α* and *Ifn‐g*) (Figure 2E), and aggravated histopathological damage (Figure 2F). Furthermore, S100A8/A9^hi^ BMDM transfer markedly aggravated intestinal fibrosis, as evidenced by increased collagen deposition (Figure 2G), thickening collagen I‐positive muscular layer (Figure 2H), and upregulated expression of fibrosis‐associated genes (*Col1a1*, *Col6a1*, *Col6a3*, *Fn1*, and *Acta2*) (Figure 2I). Collectively, these findings demonstrate the potent fibrogenic role of S100A8/A9^hi^ macrophages in the pathogenesis of colitis‐associated intestinal fibrosis.

### 2.3 *S100a8/a9* Deficiency Attenuates the Profibrotic Activity of Macrophages in Chronic Colitis Mice

To further validate these findings, BMDMs were transfected with either *S100a9*‐targeting small interfering RNA (si*S100a9*‐BMDMs) or negative control siRNA (siNC‐BMDMs). Following transfection, the cells were adoptively transferred weekly into colitis mice using the experimental strategy described above. In vivo imaging revealed enhanced fluorescence signals in the abdomen at 1, 6, 24, and 48 h after transfer (Figure S3A). At the experimental endpoint, major organs were harvested for ex vivo imaging. Strong DiR fluorescence signals were detected in the colons of mice receiving BMDMs transfers (Figure S3B). Among other organs, prominent signals were observed in the liver and spleen, while moderate fluorescence signals were detected in the lungs; no signals were observed in the heart or kidneys (Figure S3C). Furthermore, fluorescent signals in tissue sections of the colon, liver, and spleen confirmed the successful homing of the transferred cells to the colonic tissue (Figure S3D).

First, we assessed intestinal inflammation severity. BMDMs transfected with *S100a9* siRNA displayed impaired pro‐inflammatory capacity compared with negative control siRNA‐treated BMDMs. Consequently, chronic colitis mice that received *S100a9*‐silenced BMDMs exhibited higher body weight (Figure 3A), reduced inflammatory cytokine levels (*Il‐6* and *Tnf‐α*, but not *Il‐1b*; Figure 3B), longer colon length (Figure 3C), and milder pathological damage (Figure 3D). We then assessed the impact on intestinal fibrosis. Chronic colitis mice receiving si*S100a9*‐BMDMs exhibited significantly reduced fibrosis compared to siNC‐BMDM recipients, as indicated by substantial downregulation of collagens, fibronectin, and α‐SMA expression (Figure 3E,F). Histological analysis further revealed markedly decreased collagen deposition (Figure 3G) and a notable reduction in α‐SMA‐positive muscular layer thickening (Figure 3H). Collectively, these findings demonstrate that S100A8/A9^hi^ macrophages are critical drivers of colitis‐associated intestinal fibrogenesis.

**FIGURE 3:**
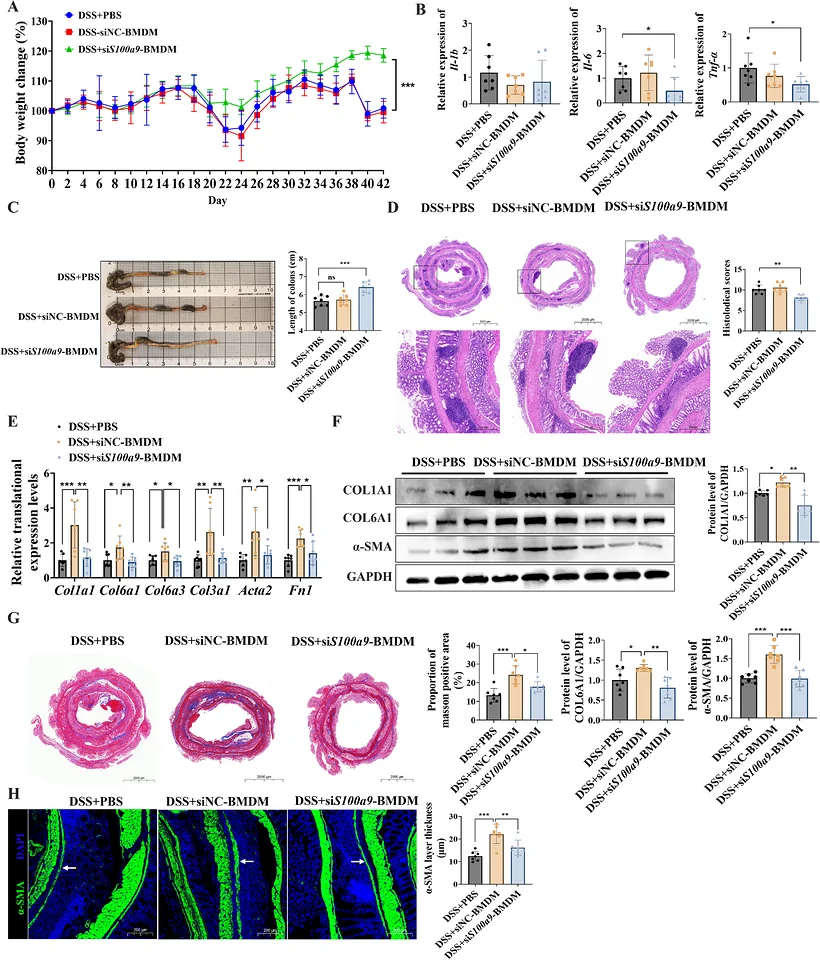
Macrophages with S100A8/A9 deficiency exhibit impaired pro‐fibrotic capacity in mice with chronic DSS‐induced colitis. Small interfering RNA (siRNA) was transfected into BMDMs for *S100a9* silencing. During chronic colitis induction with three cycles of 2% DSS, WT mice received weekly tail vein injections of 200 µL PBS or 2×10^6^ BMDMs transfected with siNC or si*S100a9* (*n* = 7/group). (A) Body weight changes presented as a percentage of the initial body weight. (B) mRNA expression of inflammatory cytokines (*Il1b*, *Il6*, and *Tnf‐α*) in colonic tissues by RT‐qPCR. (C) Representative images of colons and quantification of colon length. (D) Representative H&E staining images and corresponding histological scores of colonic tissues. Scale bar: 2000 µm (low‐magnification) and 500 µm (high‐magnification). (E) mRNA expressions of fibrosis‐associated genes (*Col1a1*, *Col6a1*, *Col6a3, Col3a1, Acta2*, and *Fn1*) in the colons were measured by RT‐qPCR. (F) Protein levels of COL1A1, COL6A1, and α‐SMA were assessed by Western blot. (G) Masson's trichrome staining was performed to quantify collagen deposition. Scale bar: 2000 µm. (H) The thickness of α‐SMA^+^ layers was determined by immunofluorescence staining. Scale bar: 200 µm. All values are expressed as mean ± SD. ns, no significant difference, ^*^ *p* < 0.05, ^**^ *p* < 0.01, ^***^ *p* < 0.001.

### 2.4 Targeting S100A8/A9 Attenuates Intestinal Fibrosis in Chronic Murine Colitis Model

Given the pivotal role of S100A8/A9^hi^ macrophages in intestinal fibrosis pathogenesis, we sought to evaluate the therapeutic potential of pharmacological S100A8/A9 inhibition. Chronic colitis was induced in WT mice with three cycles of 2% DSS insult. Starting from the second cycle, mice were treated daily via oral gavage with either the S100A8/A9 inhibitor Paquinimod (PAQ) at doses of 1, 5, or 10 mg/kg/d, or a vehicle control (Figure 4A). We first assessed the infiltration of S100A8/A9^+^ macrophages in colonic tissues using immunofluorescence and flow cytometry. S100A8/A9^+^ macrophage populations significantly increased following DSS induction; however, this expansion was markedly reduced by PAQ treatment at 10 mg/kg/d (Figure 4B,C). Correspondingly, PAQ administration (10 mg/kg/d) significantly ameliorated colonic inflammation in colitis mice across multiple parameters, as evidenced by mitigated body weight loss and colon shortening (Figure S4A,B), suppressed expressions of inflammatory cytokines (*Il‐1b, Tnf‐α, S100a8, S100a9*, and *Ccl6*; Figure S4C), and diminished histological damage (Figure 4D). Intestinal fibrosis was further evaluated through Masson's trichrome staining, α‐SMA immunofluorescence, and quantification of ECM components. All three PAQ dosages significantly ameliorated fibrosis to varying degrees, with the high‐dose (10 mg/kg/d) group exhibiting the most pronounced effects. Specifically, this was manifested as markedly reduced collagen deposition (Figure 4E), reduced thickening of the α‐SMA‐positive muscular layer (Figure 4F), and suppressed expression of ECM components, including collagen I, collagen VI, and fibronectin (Figure 4G,H).

**FIGURE 4:**
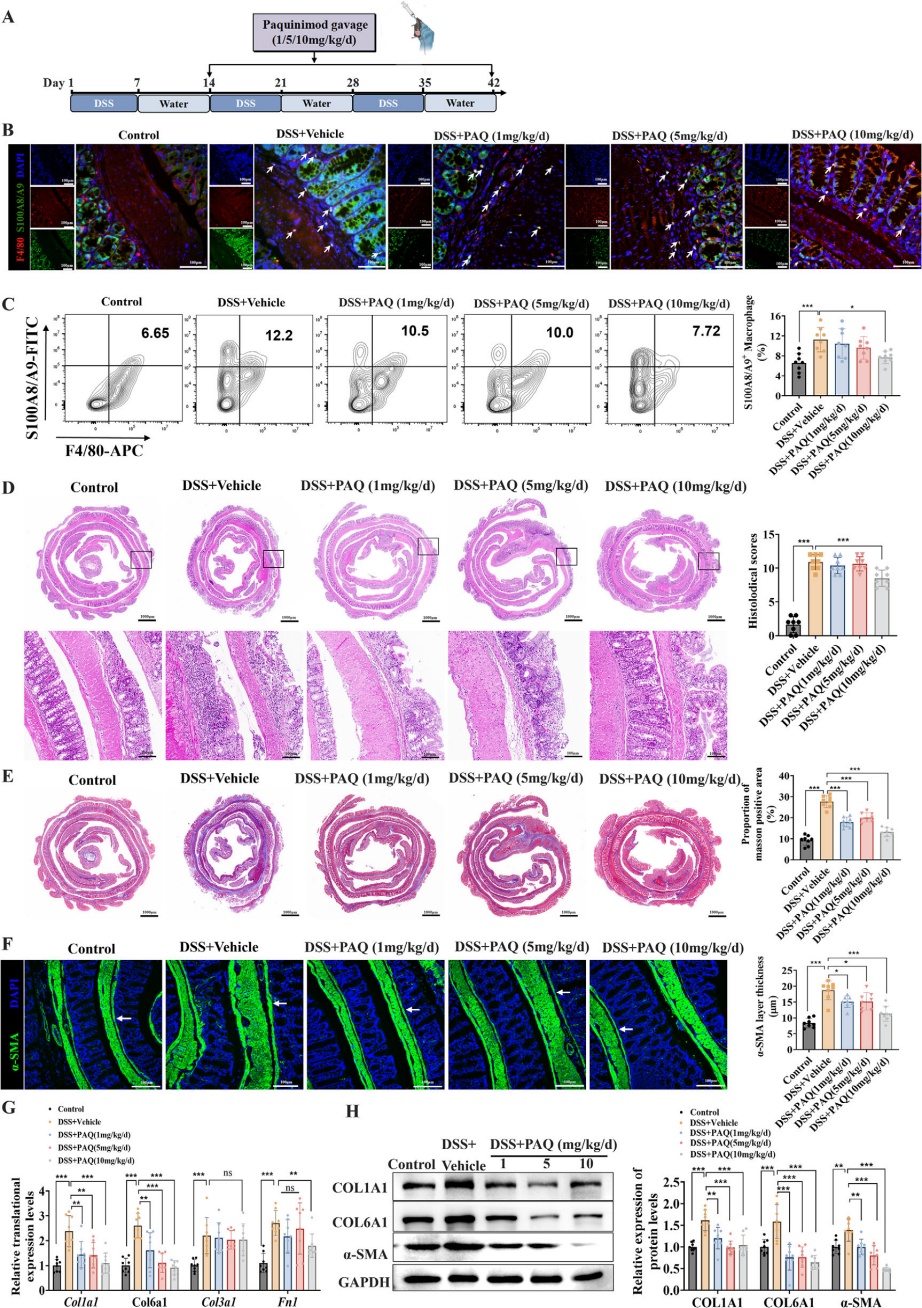
S100A8/A9 serves as an effective therapeutic target for alleviating intestinal fibrosis in a murine model of DSS‐induced colitis. WT mice were challenged with three cycles of 2% DSS and randomly assigned to the following groups receiving different doses of Paquinimod (PAQ) (*n* = 8/group): (1) Control group: received normal drinking water; (2) DSS+Vehicle group: colitis mice received 200 µL of vehicle via oral gavage; (3) DSS+PAQ (1 mg/kg/d), (4) DSS+PAQ (5 mg/kg/d), and (5) DSS+PAQ (10 mg/kg/d) groups: DSS‐induced colitis mice received PAQ orally at dosages of 1, 5, or 10 mg/kg/d, respectively, starting from the second DSS cycle. All mice were sacrificed on day 42. (A) Schematic diagram of the PAQ treatment regimen in the murine colitis model. (B) Immunofluorescence staining showing the infiltration of S100A8/A9^+^ macrophages in colonic tissues. Scale bar: 100 µm. (C) The proportion of S100A8/A9^+^ macrophages in murine colonic lamina propria mononuclear cells as measured by flow cytometry. (D) Assessment of histological damage in colonic tissues via H&E‐staining. Scale bar: 1000 µm (low magnification) and 100 µm (high magnification). (E) Representative images and quantification of collagen‐positive areas via Masson's trichrome staining. Scale bar: 1000 µm. (F) Measurement of α‐SMA‐positive layer thickness in colonic tissues by immunofluorescence. Scale bar: 100 µm. (G) mRNA levels of fibrosis‐associated genes (*Col1a1*, *Col6a1*, *Col3a1*, and *Fn1*) were measured by RT‐qPCR. (H) Protein levels of COL1A1, COL6A1, and α‐SMA were evaluated by Western blot. All values are expressed as mean ± SD. ns, no significant difference, ^*^ *p* < 0.05, ^**^ *p* < 0.01, ^***^ *p* < 0.001.

To evaluate the safety profile of PAQ, we monitored hematological parameters and hepatorenal function in the experimental mice. PAQ was well‐tolerated, with no drug‐related mortality observed throughout the study. While the 10 mg/kg/d dose of PAQ caused mild alterations in hematological parameter counts (monocyte, lymphocyte, and neutrophil) and slight elevations in transaminase levels, all values remained within normal physiological ranges (Table S4), supporting the favorable safety of PAQ. Collectively, these data demonstrate that targeting S100A8/A9 represents both a safe and efficacious therapeutic strategy for the treatment of colitis‐associated intestinal fibrosis.

### 2.5 S100A8/A9^hi^ Macrophages Drive the Activation and Extracellular Matrix Production of Fibroblasts

To investigate the mechanistic basis by which S100A8/A9^hi^ macrophages promote fibrosis, we established an in vitro co‐culture system. Mouse embryonic fibroblasts (NIH‐3T3) were incubated with BMDMs at a 2:1 ratio of 48 h (Figure 5A). EdU proliferation assays revealed that co‐culture with S100A8/A9^hi^ BMDMs significantly enhanced fibroblast proliferation compared to co‐culture with NC‐BMDMs (Figure 5B). Furthermore, S100A8/A9^hi^ BMDMs potently induced fibroblast activation and promoted the production of key ECM components, including collagen I, VI, and fibronectin (Figure 5C,D). Conversely, *S100a9* knockdown in BMDMs (which disrupts the formation of the S100A8/A9 protein complex) significantly impaired their fibrogenic capacity. Specifically, EdU assays revealed reduced fibroblast proliferation upon co‑culture with si*S100a9*‑BMDMs (Figure 5E,F). This reduction in proliferation was accompanied by attenuated fibroblast activation and significantly lower expression levels of fibronectin and multiple collagens (Figure 5G,H). Together, these results demonstrate that S100A8/A9^hi^ macrophages promote intestinal fibrosis by directly driving fibroblast proliferation, activation, and ECM synthesis.

**FIGURE 5:**
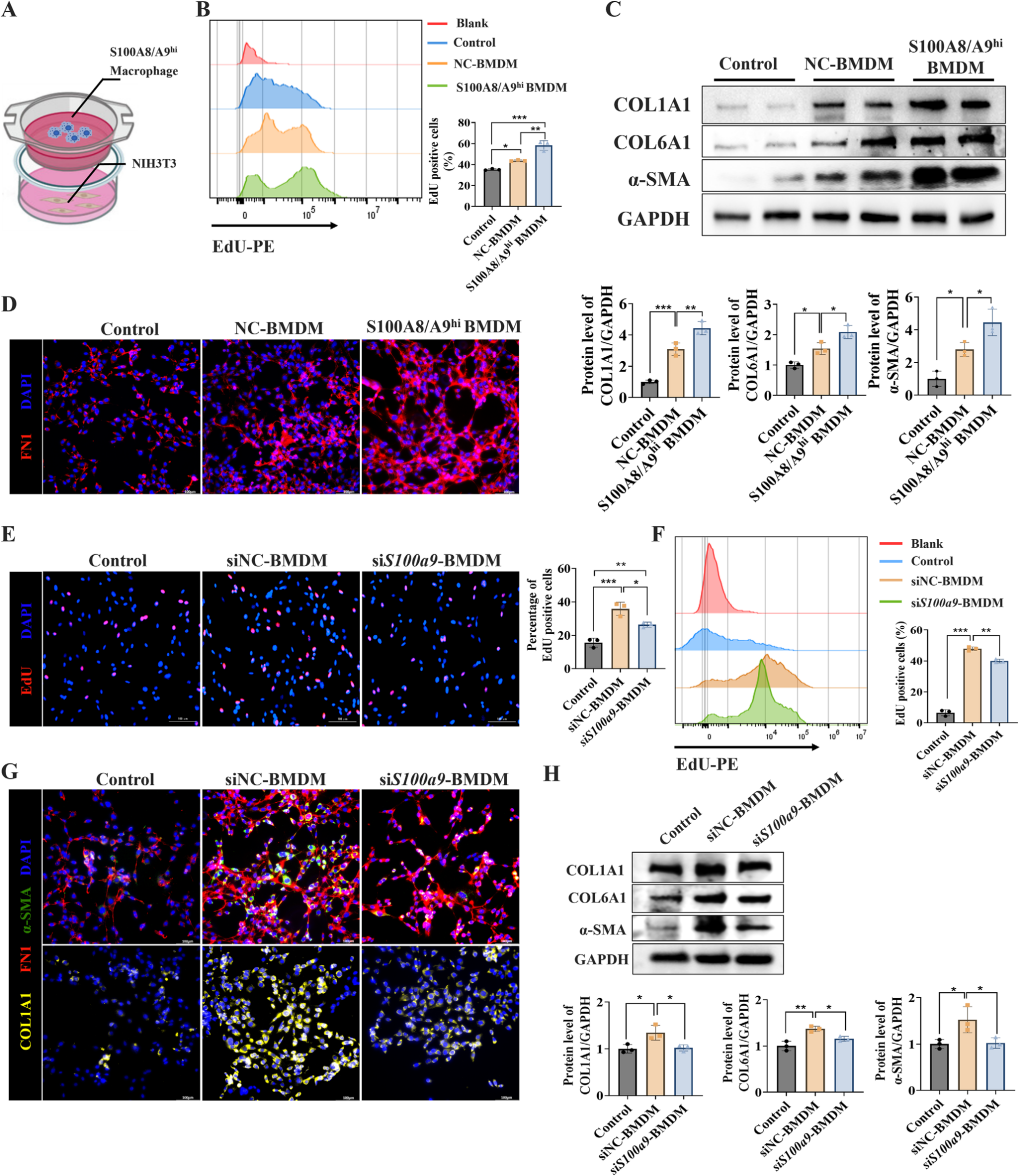
S100A8/A9^hi^ macrophages drive fibroblast proliferation, activation, and collagen production. BMDMs were isolated from WT mice. NC‐BMDMs or S100A8/A9^hi^ BMDMs were co‐cultured with the mouse embryonic fibroblasts (NIH‐3T3) at a 2:1 ratio for 48 h. (A) Schematic diagram of the co‐culture system. (B) Proliferating fibroblasts were detected by EdU staining and quantified by flow cytometry. (C) Protein levels of COL1A1, COL6A1, and α‐SMA in NIH‐3T3 cells were analyzed by Western blot. (D) Fibronectin production was assessed by immunofluorescence. Scale bar: 100 µm. BMDMs were transfected with siRNA for *S100a9* knockdown. siNC‐BMDMs or si*S100a9*‐BMDMs were co‐cultured with NIH‐3T3 cells at a 2:1 ratio for 48 h. Fibroblast proliferation was detected by EdU immunofluorescence assay (E) (scale bar: 100 µm) and flow cytometry (F). (G,H) Fibroblast activation and ECM component production (α‐SMA, fibronectin, COL1A1, and COL6A1) were assessed by immunofluorescence (G; Scale bar: 100 µm) and Western blot (H). Data are from three independent biological replicates. All values are expressed as mean ± SD. ns, no significant difference, ^*^ *p* < 0.05, ^**^ *p* < 0.01, ^***^ *p* < 0.001.

### 2.6 S100A8/A9^hi^ Macrophages Exacerbate Intestinal Fibrosis Through High Expression of Chemokine Mouse CCL6

To identify the key pro‐fibrotic mediator secreted by S100A8/A9^hi^ macrophages, we performed proteomic analysis of conditioned media from control and *S100a9*‑knockdown BMDMs. A total of 64 differentially expressed proteins were identified, including 23 upregulated and 41 downregulated proteins (Figure S5A). Kyoto Encyclopedia of Genes and Genomes (KEGG) analysis revealed significant enrichment in the ECM‐receptor interaction pathway, which is critically linked to fibrogenesis (Figure 6A). Among the most significantly altered proteins, we prioritized the chemokine mouse CCL6 (mCCL6) for further investigation, given its secretory nature and established role in organ fibrosis (Figure 6B,C) [21, 22].

**FIGURE 6:**
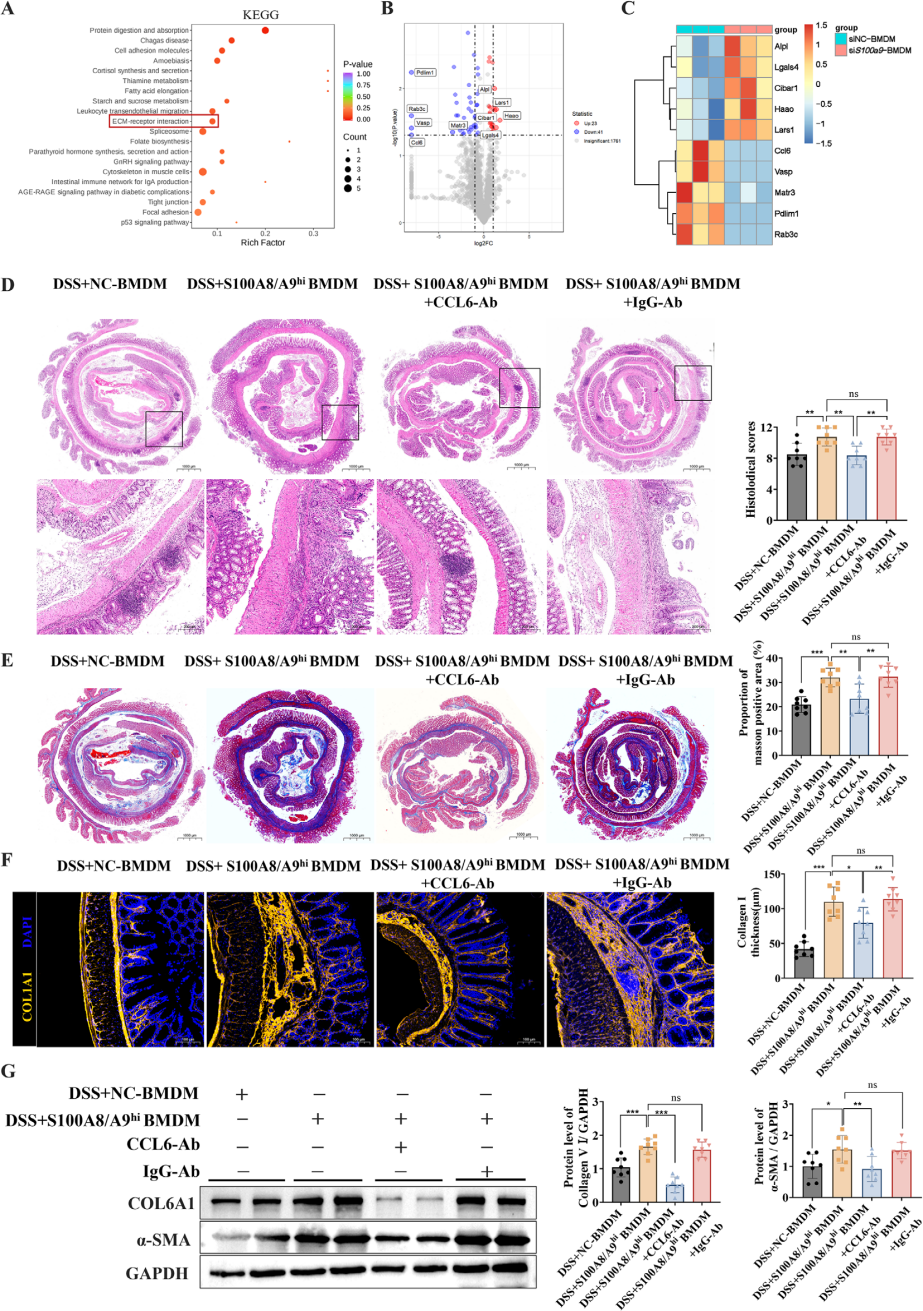
S100A8/A9^hi^ macrophages drive colitis‐associated intestinal fibrosis via the mCCL6 ligand. BMDMs were transfected with *S100a9‐*targeting siRNA to disrupt the S100A8/A9 heterodimer and subsequently cultured in serum‐free medium for 48 h. Supernatants were collected for proteomics profiling (*n* = 3 per group). For in vivo studies, DSS‐induced colitis mice received adoptive transfer of either NC‐BMDMs or S100A8/A9^hi^ BMDMs. A subset of mice receiving S100A8/A9^hi^ BMDMs were also administered intraperitoneal injections of either a CCL6‐neutralizing antibody or an isotype control antibody (4 µg/ mouse), starting from the first DSS cycle and repeated every three days (*n* = 8/group). (A) Kyoto Encyclopedia of Genes and Genomes (KEGG) pathway analysis of differentially expressed proteins in BMDMs following *S100a9* knockdown. (B,C) Volcano plot (B) and hierarchical clustering analysis (C) highlighting the top five upregulated and downregulated proteins. (D) Representative images and histological scoring of colonic tissues via H&E staining. Scale bar: 1000 µm (low magnification) and 200 µm (high magnification). (E) Masson's trichrome staining and quantification of collagen deposition in the colon. Scale bar: 1000 µm. (F) Immunofluorescence staining and quantification of collagen I‐positive layer thickness. Scale bar: 100 µm. (G) Protein expression levels of COL6A1 and α‐SMA determined by Western blot. All values are expressed as mean ± SD. ns, no significant difference, ^*^ *p* < 0.05, ^**^ *p* < 0.01, ^***^ *p* < 0.001.

To interrogate the functional contribution of mCCL6 to the profibrotic activity of S100A8/A9^hi^ macrophages, we administered a neutralizing anti‐CCL6 antibody to DSS‐induced colitis mice that had received S100A8/A9^hi^ BMDMs transfers. Antibody treatment effectively abrogated the elevated mCCL6 levels in both colon tissue and serum induced by the cell transfer (Figure S5B,C). Consequently, mCCL6 neutralization significantly mitigated the exacerbation of intestinal inflammation, as evidenced by improved body weight (Figure S5D), alleviation of colon shortening (Figure S5E), reduced expression of inflammatory cytokines (*Il‐1b, Il‐6*, and *Tnf‐α*,) (Figure S5F), and diminished pathological damage (Figure 6D). Importantly, pharmacological blockade of mCCL6 markedly improved intestinal fibrosis, as evidenced by decreased collagen deposition (Figure 6E), reduced thickening of the collagen I‐positive muscular layer (Figure 6F), and downregulation of fibrosis‐associated genes (*Col1a1*, *Col6a1*, *Col6a3*, and *Col3a1*) (Figure S5G), as well as reduced protein levels of COL6A1 and α‐SMA (Figure 6G).

In vitro experiments further supported these findings; adding an anti‐CCL6 neutralizing antibody to the co‑culture system impaired the ability of S100A8/A9^hi^ BMDMs to promote fibroblast proliferation (Figure S6A,B), activation, and ECM production, including collagen I and VI (Figure S6C,D). Moreover, the CCL6 receptor, CCR1, was found to be upregulated in co‐cultured fibroblasts at both the mRNA and protein levels (Figure S7A–C). Consequently, treatment with a specific CCR1 inhibitor abolished the enhanced fibroblast proliferation and ECM production induced by S100A8/A9^hi^ BMDM‐derived mCCL6 (Figure S7D,E). Furthermore, the migration and contraction abilities of the co‐cultured fibroblasts were weakened under CCR1 blockade (Figure S7F,G). Together, these findings demonstrate that mCCL6 serves as a critical downstream mediator through which S100A8/A9^hi^ macrophages drive colitis‐associated fibrosis via CCR1 signaling.

### 2.7 S100A8/A9^hi^ Macrophages Drive CCL6 Production Through STAT3 Activation

To investigate the mechanisms regulating CCL6 production in S100A8/A9^hi^ macrophages, we performed an integrative intersection analysis using three transcription factor databases (JASPAR, TFBs, and KnockTF), identifying nine potential regulators (*Atoh1, Bcl6, Elf5, Meis1, Myb, Myc, Sox11, Stat3* and *Stat4*) (Figure 7A). Protein‐protein interaction analysis with the STRING database revealed a strong and specific association between S100A9 and STAT3 among these candidates (Figure 7B). Given the established roles of S100A9 in regulating STAT3 in other pathological contexts [23, 24], we sought to validate this relationship in our system. Initially, STAT3 phosphorylation significantly increased upon stimulation with recombinant S100A8‐S100A9 protein (Figure 7C). Furthermore, we investigated the mechanism by which S100A8/A9 activates STAT3. S100A8/A9 has been identified as a damage‐associated molecular pattern that signals through the cell‐surface receptors Toll‐like receptor 4 (TLR4) and the receptor for advanced glycation end‐products (RAGE) [25, 26]; therefore, we examined whether S100A8/A9 activates STAT3 via TLR4 or RAGE by using specific inhibitors. We found that TLR4 inhibition significantly attenuated both p‐STAT3 levels and CCL6 secretion, whereas RAGE inhibition had no discernible effect (Figure 7D,E). Moreover, co‐localization of S100A8/A9 and TLR4 was reduced upon TLR4 inhibition (Figure 7F). Collectively, these data demonstrate that S100A8/A9 induces STAT3 activation through TLR4 signaling.

**FIGURE 7:**
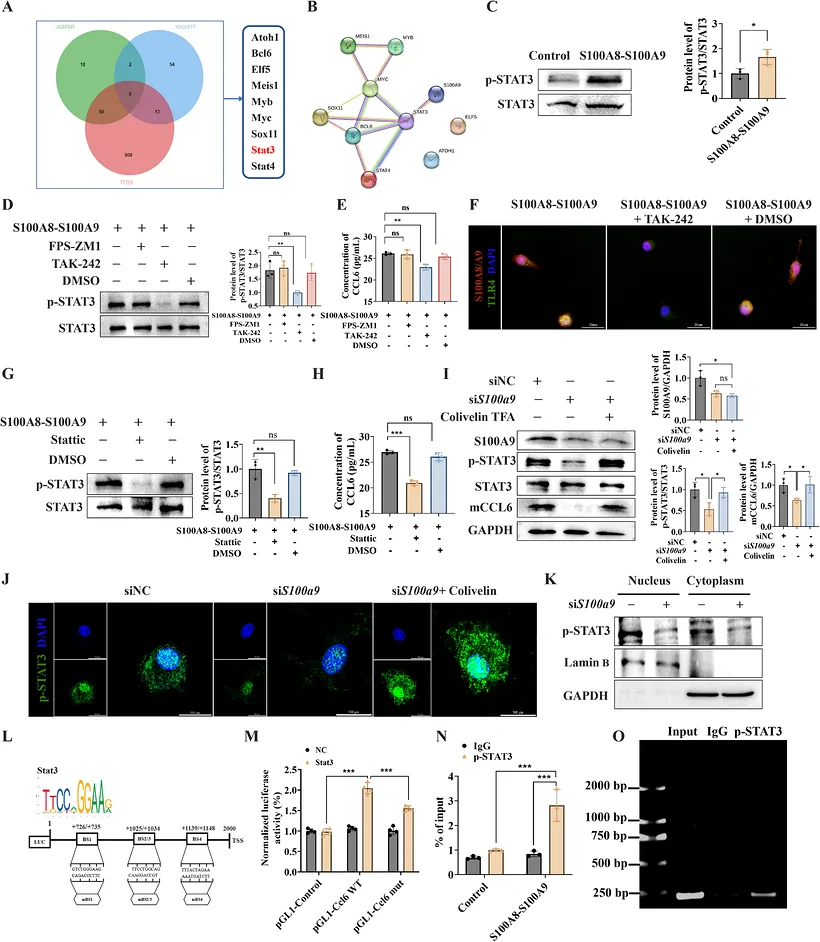
S100A8/A9^hi^ macrophages mediate CCL6 expression through activation of the transcription factor STAT3. (A) Venn diagram integrating multiple bioinformatic databases to identify potential upstream transcription factors regulating *Ccl6* expression. (B) Protein‐protein interaction network between the nine transcription factors and S100A9. (C–F) BMDMs were stimulated with recombinant S100A8‐S100A9 protein (1 µg/mL) with or without pretreated with FPS‐ZM1 (1 µm), TAK‐242 (1 µm) or DMSO vehicle for 1 h. p‐STAT3 and total STAT3 were assessed by Western blot at 2 h (C and D), and CCL6 levels in culture supernatants were measured by ELISA at 24 h (E). The colocalization of S100A8/A9 with TLR4 in BMDMs was assessed by immunofluorescence staining (F); Scale bar, 20 µm. (G,H) Following pretreatment with or without Stattic (5 µm) for 1 h, BMDMs were stimulated with recombinant S100A8‐S100A9 protein (1 µg/mL). p‐STAT3 and total STAT3 levels were assessed by Western blot at 2 h (G), and CCL6 in culture supernatants was quantified by ELISA at 24 h (H). (I,J) BMDMs were transfected with siNC or si*S100a9*, followed by treatment with Colivelin TFA (50 µg/mL) for 4 h to activate STAT3. Protein levels of S100A9, p‐STAT3/STAT3, and CCL6 were measured by Western blot (I), and p‐STAT3 expression was visualized by immunofluorescence (J; Scale bar: 500 µm). (K) Western blot analysis of p‐STAT3 levels in nuclear and cytoplasmic fractions of BMDMs transfected with siNC or *siS100a9*. (L) Schematic representation of putative STAT3 binding sites within the *Ccl6* promoter. (M) Dual‐luciferase reporter assays were performed in HEK 293T cells co‐transfected with a control vector (NC) or a *Stat3* expression plasmid, together with reporter vectors pGL1‐Control, pGL1‐Ccl6 wild‐type (WT), or pGL1‐Ccl6 mutant (mut). Promoter activity was measured and normalized. N, O) BMDMs were treated with recombinant S100A8‐S100A9 protein (1 µg/mL) for 2 h. STAT3 recruitment to the *Ccl6* promoter was analyzed by chromatin immunoprecipitation (ChIP) assay. The enrichment of p‐STAT3 at the promoter region was quantified by RT‐qPCR and expressed as a percentage of the total input (N). Representative agarose gel images confirmed the specificity of the PCR amplification (O). All values are expressed as mean ± SD. ns, no significant difference, ^*^ *p* < 0.05, ^**^ *p* < 0.01, ^***^ *p* < 0.001.

We next examined whether S100A8/A9 induces CCL6 production via STAT3 activation. Notably, pharmacological inhibition of STAT3 with Stattic markedly attenuated CCL6 secretion from S100A8‐S100A9‐stimulated macrophages (Figure 7G,H). To substantiate these findings under endogenous conditions, we examined whether intrinsic S100A8/A9 expression similarly governs the STAT3‐CCL6 axis. siRNA‐mediated knockdown of *S100a9* in BMDMs successfully reduced S100A9 expression, concomitantly decreasing both STAT3 phosphorylation and CCL6 protein levels. Importantly, Colivelin TFA‐mediated STAT3 activation rescued the CCL6 suppression caused by *S100a9* knockdown (Figure 7I). To further confirm the S100A9‐STAT3 axis, we examined STAT3 phosphorylation levels in both cytoplasmic and nuclear fractions of BMDMs. *S100a9* knockdown diminished p‐STAT3 levels in both compartments, as shown by confocal immunofluorescence microscopy and Western blotting; this suppression was effectively reversed by the addition of a STAT3 activator (Figure 7J,K). Next, we examined whether STAT3 directly regulates CCL6 transcription. Based on JASPAR database predictions, we identified four classical STAT3‐binding motifs within the *Ccl6* promoter (Figure 7L). Dual‐luciferase reporter assays using wild‐type (WT) and mutant (mut) constructs confirmed that STAT3 significantly enhanced the transcriptional activity of the WT *Ccl6* promoter, an effect that was abolished upon mutation of the binding sites (Figure 7M). Additionally, chromatin immunoprecipitation (ChIP) assays verified that STAT3 binding to the Ccl6 promoter was markedly enhanced following S100A8‐S100A9 stimulation (Figure 7N,O). Collectively, these results demonstrate that CCL6 production in S100A8/A9^hi^ BMDMs is driven by STAT3 activation.

### 2.8 The human Ortholog hCCL15 Exerts Comparable Pro‐Fibrotic Effects on Fibroblasts Through CCR1

The functional human orthologs of murine CCL6 are CCL15 (hCCL15) and CCL23 (hCCL23) [27]. According to the Human Protein Atlas data, hCCL15 is highly expressed in the intestinal tract, whereas hCCL23 shows low and non‐specific expression (Figure S8A). In clinical samples, hCCL15 expression was markedly upregulated in stenotic regions and exhibited notable co‐localization with collagen deposits (Figure 8A,B), while no significant difference was observed for CCL23 (Figure S8B,C). Systemically, hCCL15 levels in the serum were significantly elevated in CD patients presenting with intestinal strictures relative to patients without strictures (Figure 8C). Transcriptomic analysis further revealed a strong positive correlation between *CCL15* expression and several collagen‐related genes (*COL1A1*, *COL3A1*, and *FN1)*, though not with *COL6A1* (Figure 8D). Consequently, we focused on hCCL15 for subsequent analyses. To assess direct fibrogenic effects, we isolated primary human intestinal fibroblasts (HIFs) from stenotic regions of CD patients and treated them with recombinant hCCL15 (0, 5, 10, and 20 ng/mL) for 48 h. Both 10 and 20 ng/mL concentrations significantly promoted COL1A1 synthesis in fibroblasts; however, no further increase was observed at the higher dose (Figure 8E). Therefore, 10 ng/mL hCCL15 was selected for further investigation. As expected, hCCL15 promoted HIFs migration in wound healing assays (Figure 8F). In addition, hCCL15 enhanced HIFs activation and proliferation, as evidenced by increased α‐SMA and Ki67 expression via immunofluorescence staining (Figure 8G). Furthermore, CCL15 stimulation significantly upregulated the mRNA expression of fibrosis‐associated genes (*ACTA2*, COL1A1, *COL3A1*, *COL6A1*, *COL6A3*, and *FN1*) in fibroblasts (Figure 8H).

**FIGURE 8:**
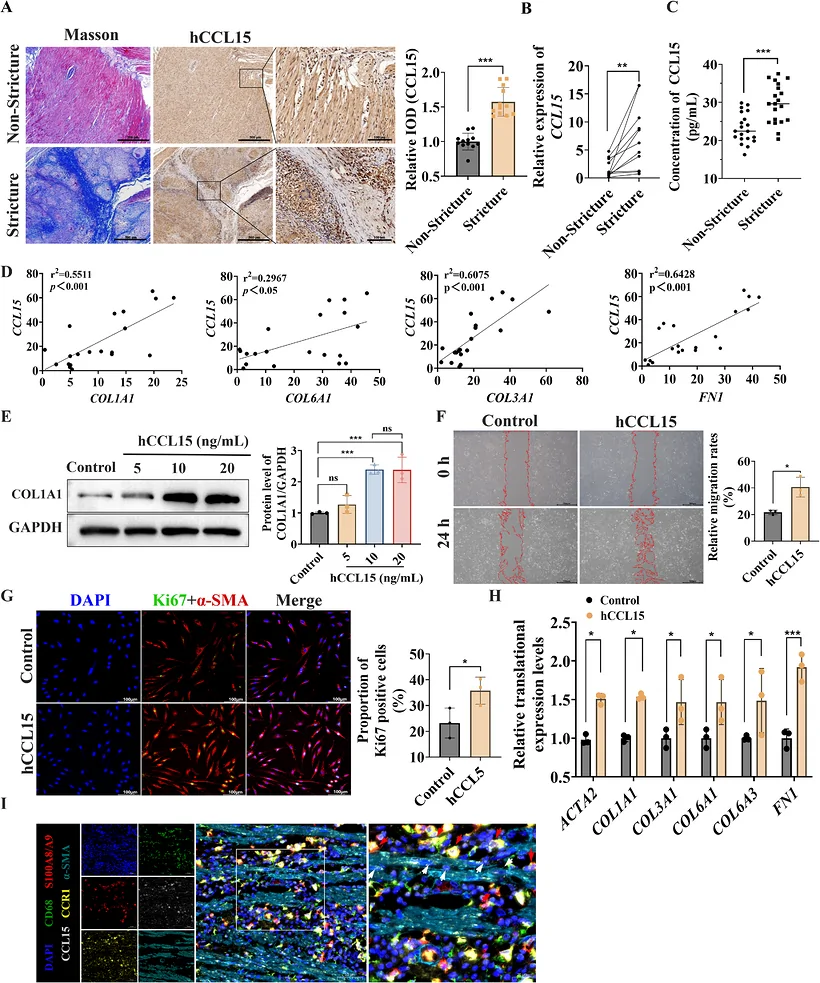
Human CCL15 promotes the activation, proliferation, and migration of human intestinal fibroblasts. (A) Representative images of Masson's trichrome staining and IHC showing fibrosis levels and hCCL15 expression in stenotic and non‐stenotic intestinal surgical specimens from patients with CD. Scale bar: 500 µm (low magnification) and 100 µm (high magnification). *n* = 4 patients; 3 fields per zone. (B) Relative *CCL15* mRNA abundance in stricture versus non‐stricture regions was quantified by RT‐qPCR. (C) Serum concentrations of hCCL15 in CD patients with or without intestinal strictures were detected using ELISA. (D) Correlation analysis between *CCL15* and collagen‐associated genes (*COL1A1, COL6A1, COL3A1*, and *FN1*) in CD intestines. (E) Human intestinal fibroblasts (HIFs) isolated from stenotic regions were treated with recombinant CCL15 (5,10, and 20 ng/mL) for 48 h, and COL1A1 protein levels were then assessed by Western blot. (F–H) HIFs were stimulated with hCCL15 (10 ng/mL) for 48 h. Migration was assessed by wound healing assay (F; Scale bar: 500 µm). α‐SMA and Ki67 immunofluorescence staining was performed to evaluate activation and proliferation, and Ki67+ cells were quantified (G; Scale bar: 100 µm). mRNA levels of *ACTA2*, *COL1A1*, *COL3A1*, *COL6A1*, *COL6A3*, and *FN1* were measured by RT‐qPCR (H). I) Immunofluorescence co‐localization analysis of S100A8/A9^+^ CCL15^+^ macrophages and CCR1^+^ α‐SMA^+^ fibroblasts in intestinal stricture sections from patients with CD. Red arrows indicate S100A8/A9^+^ CCL15^+^ macrophages located within 30 µm of the nearest fibroblast; white arrows indicate CCR1^+^ fibroblasts. Scale bars: 50 µm (low magnification) and 20 µm (high magnification). *n* = 4 patients; 3 fields per zone. All values are expressed as mean ± SD. ns, no significant difference, ^*^ *p* < 0.05, ^**^ *p* < 0.01, ^***^ *p* < 0.001.

Since hCCL15 signals through the receptors CCR1 and CCR3 [28, 29], we first examined the expression levels within the fibroblast subset of a full‐thickness CD scRNA‐seq dataset. *CCR1* exhibited a higher transcriptional level than *CCR3* (Figure S8D). This expression pattern was consistently validated in paired non‐stenotic and stenotic tissues from CD patients, as well as in primary fibroblasts isolated from these matched regions (Figure S8E,F). Importantly, immunofluorescence staining revealed that S100A8/A9^+^ CCL15^+^ macrophages were spatially adjacent to CCR1^+^ fibroblasts (Figure 8I), suggesting CCL15‐CCR1 signaling between S100A8/A9^hi^ macrophages and fibroblasts in stenotic regions of CD. Given the predominant expression of CCR1, we hypothesized that it mediates the pro‐fibrotic effects of hCCL15. To test this, HIFs were pretreated with the CCR1‐specific antagonist BX471 for 6 h prior to hCCL15 stimulation. CCR1 blockade significantly attenuated the key pro‐fibrotic responses: collagen production was reduced (Figure S8G), cell migration was impaired (Figure S8H), and fibroblast proliferation and activation were markedly decreased (Figure S8I). These results demonstrate that hCCL15, the human ortholog of murine CCL6, contributes to intestinal fibrosis in CD by directly promoting the fibroblasts activation, proliferation, migration, and collagen synthesis via the CCR1 receptor.

## 3 Discussion

Our study identified a novel profibrotic S100A8/A9^hi^ macrophage subset that drives CD‐associated intestinal fibrosis. Mechanistically, CCL6 emerged as the key profibrotic mediator produced by S100A8/A9^hi^ macrophages in a STAT3‐dependent manner. Furthermore, pharmacological inhibition of S100A8/A9 or neutralization of mCCL6 robustly suppressed fibrotic progression, underscoring the therapeutic potential of targeting this macrophage population. Finally, hCCL15, the human ortholog of mCCL6, was proved to be a potent profibrotic mediator that drives fibroblast activation via CCR1, highlighting its potential as a therapeutic target for intestinal fibrosis.

*S100A8* and *S100A9* are members of the S100 calcium‐binding protein family that form a constitutively expressed and functionally active heterodimer, predominantly in granulocytes, monocytes, and early‐differentiation macrophages [30, 31]. Recently, a unique pro‐inflammatory S100A8/A9^hi^ macrophage subset was identified by scRNA‐seq in a mouse acute kidney injury (AKI) model, and this finding was further supported by the correlation between S100A8/A9^+^ macrophage infiltration and renal dysfunction in human AKI [32]. In our study, the adoptive transfer of S100A8/A9^hi^ macrophages significantly aggravated colonic inflammation in mice, which further validated its pro‐inflammatory role. Macrophages are consistently found in spatial proximity to collagen‐producing myofibroblasts [33, 34], and have long been implicated as key drivers of fibrogenesis [35, 36, 37]. As previously confirmed, colonic fibrosis in CD patients is consistently associated with dense infiltrates of CD68^+^ monocytes/macrophages [38, 39]. ScRNA‐seq data revealed an expansion of S100A8/A9^+^ macrophages in liver fibrotic mice and showed that inhibiting their differentiation significantly attenuated fibrosis [18]. Consistently, S100A8/A9^+^ macrophages were also found to be enriched in cirrhotic patients and were shown to possess pro‐fibrotic functions [40]. Moreover, a specific population of monocyte‐derived S100A9‐high macrophages has been proved to mediate both inflammation and fibrosis during myocardial ischemia‐reperfusion [41]. In line with these previous findings, we identified a distinct S100A8/A9^hi^ macrophage subset enriched in the stenotic intestinal regions of CD and adjacent to fibroblasts. Adoptive transfer of these macrophages exacerbated colonic fibrosis in colitis mice and promoted proliferation, activation, and ECM production in co‐cultured fibroblasts, collectively confirming their profibrotic role in CD.

Quinoline‐3‐carboxamides (Q compounds) are recognized as immunomodulatory agents that exhibit therapeutic potential in a range of preclinical models, including inflammatory diseases [42, 43, 44, 45] and malignancy [46, 47]. Paquinimod (PAQ) is such a Q compound that can actively bind to S100A8/A9 compounds [48], which has been reported to prevent the development of diabetes in the non‐obese diabetic mice [49], ameliorate pathology in collagenase‐induced osteoarthritis models [50], alleviate coronavirus‐induced lung injury [51], reverse established murine liver fibrosis [52] and mitigate skin fibrosis in a systemic sclerosis experimental model [53]. In addition, blockade of S100A8/A9 with PAQ significantly reduced the number of infiltrated S100A9^+^ macrophages in murine AKI models and significantly alleviated the AKI injury [32]. In our chronic colitis models, oral administration of PAQ (10 mg/kg/d) markedly reduced S100A8/A9^+^ macrophage infiltration, which consequently mitigated both inflammatory injury and intestinal fibrosis. The treatment was well‐tolerated, with no mortality, drug‐induced cytopenia, or impairment of hepatic and renal function, underscoring its promise as a safe treatment for intestinal fibrosis in CD.

Murine CCL6, primarily secreted by macrophages and epithelial cells [54], is implicated in various pathological states, including pulmonary fibrosis [21], experimental demyelinating diseases [55], and eosinophilic airway inflammation [56]. In murine colitis, elevated CCL6 recruits dendritic cells and macrophages, thereby exacerbating colonic inflammation [57, 58]. CCL6 has been thought to exert its effects through CCR1 activation [56]. Therapeutic strategies targeting mCCL6 or its receptor CCR1 effectively ameliorate fibrotic pathology by preventing immune cell recruitment [22, 59]. Our proteomic profiling identified mCCL6 as the key profibrotic mediator from S100A8/A9^hi^ macrophages, orchestrating fibroblast proliferation, activation, and ECM production. These effects were abolished by mCCL6 blockade or CCR1 inhibition in both co‐culture systems and murine models. Crucially, we translated these findings to human pathology by identifying hCCL15 as the functional ortholog. The marked upregulation of hCCL15 in stenotic CD tissues and its strong correlation with ECM‐related genes (*COL1A1*, *COL3A1*, and *FN1*) suggest that this axis is highly conserved and clinically relevant. As anticipated, hCCL15 exhibited profibrotic effects, evidenced by increased fibroblast proliferation, migration, activation, and collagen synthesis. Importantly, S100A8/A9^+^ CCL15^+^ macrophages were also found to be located adjacent to CCR1^+^ fibroblasts in stricture sections from CD patients, indicating the profibrotic regulatory function of S100A8/A9^hi^ macrophages on intestinal fibroblasts through the hCCL15‐CCR1 axis.

Collectively, our data identify the STAT3‐mediated S100A8/A9^hi^ macrophage–mCCL6/hCCL15–CCR1 axis as a major profibrotic pathway in CD. However, the current evidence relies primarily on pharmacological and adoptive transfer approaches. Lineage‐specific genetic models—such as myeloid‐specific STAT3 conditional knockout mice or S100A8/A9‐deficient bone marrow chimeras—will be essential to formally establish the macrophage‐centric mechanism, and fibroblast‐restricted genetic validation of CCR1 remains an important future direction. These studies represent critical next steps to solidify the causal role of this signaling axis in intestinal fibrosis.

## 4 Conclusion

In conclusion, our study provides the first evidence establishing S100A8/A9^hi^ macrophages as a pivotal pathogenic subset in intestinal stricture formation in CD. Pharmacological targeting of S100A8/A9 significantly attenuated intestinal fibrotic progression. Mechanistically, S100A8/A9^hi^ macrophages activate STAT3 to drive mCCL6 production, which serves as the major profibrotic mediator via CCR1 signaling on fibroblasts. Notably, its human ortholog hCCL15 exerts comparable profibrotic effects. By positioning S100A8/A9, STAT3, mCCL6/hCCL15, and CCR1 as viable therapeutic targets, this study offers a promising framework for targeted interventions against intestinal fibrosis in CD patients.

## 5 Experimental Section

### 5.1 Single‐Cell RNA Sequencing Data Analysis

We used the R package Seurat (5.3.0) and the ClusterProfiler (4.12.3) package to analyze the single‐cell RNA sequencing data generated from 13 ileal CD patients with full thickness specimens. The raw count matrix was processed with the SCTransform function, which performs regularized negative binomial regression to simultaneously normalize the data, identify highly variable genes (top 3,000 genes by default), and correct for sequencing depth and technical noise. Cell clusters were redefined into 15 distinct subpopulations by identifying the top 10 marker genes for each cluster using the FindAllMarkers function, followed by manual annotation. Cell–cell communication between fibroblasts and other cell subpopulations in the stricture condition was quantified using the computeCommunProb function from the CellChat package (2.2.0). All analyses were performed in R (version 4.4.0), and visualizations were generated using ggplot2 (version 4.0.0).

### 5.2 Single‐Cell Distance Analysis

To quantitatively assess the transcriptional heterogeneity and inter‐subgroup relationships within the myeloid compartment of CD, we performed single‐cell distance (scDIST) analysis [20]. The scDIST algorithm (v1.1.4) was used to compute the transcriptional distances between conditional means in high‐dimensional gene expression space for each cell type following group comparisons: Inflamed vs. Non‐involved and Stricture vs. Inflamed. A linear mixed‐effects model was applied to account for variability across samples and other technical factors, enabling identification of cell subpopulations with the most pronounced changes across conditions.

### 5.3 Patients and Samples Collection

Patients with CD were enrolled in The First Affiliated Hospital with Nanjing Medical University. For this study, serum samples were collected from patients with and without intestinal strictures. Surgical specimens were obtained from stenotic segments and matched non‐stenotic regions, which encompassed both non‐involved and inflamed intestinal areas. Written informed consent was obtained from all patients prior to sample collection. The Crohn's Disease Activity Index (CDAI) scores were used to control for potential confounding effects due to between‐group differences in disease severity, ensuring that any observed differences could be attributed to the presence or absence of strictures. The clinical characteristics of the study participants were summarized in Table S1.

### 5.4 Mice

WT C57BL/6 mice (aged 7 weeks, 18–20 g) were purchased from Charles River (USA) and were housed under specific pathogen‐free (SPF) conditions in the Nanjing Medical University Animal Care facility.

### 5.5 Chronic Colitis Model Induction and Experimental Grouping

To establish a chronic colitis model, mice were subjected to three cycles of 2% DSS (MP Biomedicals, USA). Each cycle consisted of 7 days of DSS exposure in drinking water followed by 7 days of recovery on regular water.

An adoptive transfer model was established to investigate the role of S100A8/A9^hi^ macrophages in colitic mice. BMDMs isolated from WT mice were stimulated with LPS (100 ng/mL, 24 h) to induce the S100A8/A9‐high phenotype. For macrophage depletion, mice received an intravenous injection of clodronate liposomes (200 µL, #40337ES10, YEASEN, China) 48 h before BMDM transfer and were then divided into four experimental groups (*n* = 8/group) on day 5: (1) Control group: Received normal drinking water throughout the experiment; (2) DSS + PBS group: Received a 200‐µL intravenous injection of PBS solution; (3) DSS + NC‐BMDM group: Received weekly intravenous injections of normal control (NC) BMDMs (2×10^6^/mouse) isolated from WT mice; (4) DSS + S100A8/A9^hi^ BMDM group: Received weekly intravenous injections of S100A8/A9^hi^ BMDMs (2×10^6^/mouse). Similarly, BMDMs transfected with siNC or si*S100a9* were adoptively transferred into recipient mice with DSS‐induced colitis following a similar protocol (*n* = 7/group).

To investigate the efficacy of S100A8/A9 inhibition in alleviating intestinal fibrosis, mice with DSS‐induced colitis were administered Paquinimod (PAQ, HY‐100442, MCE, USA) via daily gavage starting from the second DSS cycle. Mice (*n* = 8/group) were randomly assigned to the following groups: (1) Control group: received normal water; (2) DSS+Vehicle group: mice with DSS‐induced colitis received 200 µL vehicle (20 µL DMSO,80 µL PEG300, 10 µL Tween 80 and 90 µL normal saline mixed solution) daily; (3) DSS+PAQ (1 mg/kg/d), (4) DSS+PAQ (5 mg/kg/d) and (5) DSS+PAQ (10 mg/kg/d) groups: mice with DSS‐induced colitis received PAQ by daily gavage at 1, 5, or 10 mg/kg, respectively.

To elucidate the contribution of S100A8/A9^hi^ macrophage‐derived mCCL6 to colitis‐associated fibrosis, mice were divided into four experimental groups (*n* = 8/group): (1) DSS + NC‐BMDM group; (2) DSS + S100A8/A9^hi^ BMDM group; (3) DSS + S100A8/A9^hi^ BMDM+CCL6‐Ab group; (4) DSS + S100A8/A9^hi^ BMDM +IgG‐Ab group. Groups 1 and 2 received the respective BMDM transfers as described above. In addition to receiving S100A8/A9^hi^ BMDMs, mice in groups 3 and 4 received intraperitoneal injections of either a CCL6‐neutralizing antibody (4 µg/mouse, MAB487, R&D Systems, USA) or an equal dose of IgG2B isotype control (MAB0061, R&D Systems, USA). Antibody treatment began at the first DSS cycle and was repeated every three days. Body weight was monitored every 2 days throughout all the experiments. On day 42, all experimental mice were euthanized by CO_2_ inhalation (30%–70% chamber displacement rate/min) followed by cervical dislocation. Tissues were subsequently harvested for pathological and molecular analyses.

### 5.6 Macrophage Trafficking

To track the in vivo distribution of transferred macrophages, BMDMs were labeled with the lipophilic fluorescent dye DiR (5 µM; #40757ES25, YEASEN, China) for 30 min at 37°C, followed by adoptive transfer into colitic mice via tail vein injection. Mice were anesthetized with isoflurane (induction: 3%; maintenance: 1.5%–2% in oxygen), and cell homing and persistence were monitored at 1, 6, 24, and 48 h post‐injection using an In Vivo Imaging System (IVIS) at excitation/emission wavelengths of 750/780 nm. Upon completion of the study, the colon and major organs (heart, liver, spleen, lungs, and kidneys) were harvested for ex vivo fluorescence imaging.

### 5.7 Small Interfering RNA Transfection

Mature BMDMs were transfected with siRNA targeting *S100a9* (si*S100a9*) using Lipofectamine RNAiMAX (#13778030, Invitrogen, USA) in Opti‐MEM I (#31985‐070, Gibco, USA) following the manufacturer's protocol. The siRNA sequences, synthesized by Corues Biotechnology (China), are listed in Table S3. The third sequence was used for subsequent experiments.

### 5.8 Cell Culture and Treatment

Human intestinal fibroblasts (HIFs) were isolated from stenotic surgical specimens of CD patients according to established protocols [60, 61]. Both HIFs (passages 3–8) and mouse embryonic fibroblasts (NIH‐3T3) were maintained in Dulbecco's modified Eagle's medium (DMEM, Gibco, USA) supplemented with 10% fetal bovine serum (FBS; #CTCC‐002‐071, Meisen CTCC, China) and 1% penicillin‐streptomycin (NCM Biotech, China) for all experiments.

To induce an S100A8/A9‐high phenotype, BMDMs were pretreated with LPS (100 ng/mL; Sigma #297‐473‐0), IL‐1β (100 ng/mL; GenScript #Z02985), or TNF‐α (100 ng/mL; GenScript #Z02916) for 6, 12, or 24 h. The proportion of S100A8/A9‐positive BMDMs was quantified by flow cytometry following 24 h of LPS stimulation. Co‐culture experiments were performed using a Transwell system. Briefly, S100A8/A9^hi^ BMDMs were induced as described above, while another set was transfected with either siNC or si*S100a9*. NIH‐3T3 cells (#CRL‐1658, Meisen CTCC, China, 1 × 10^5^) were then co‐cultured with BMDMs (2 × 10^5^) for 48 h across the following groups: (1) NC‐BMDMs vs. S100A8/A9^hi^ BMDMs, and (2) siNC‐ vs. si*S100a9*‐BMDMs.

To evaluate the profibrotic effects of S100A8/A9^hi^ BMDM‐derived CCL6, two strategies were used: (1) BMDMs were pre‐incubated for 6 h with an anti‐CCL6 antibody (40 ng/mL; R&D Systems, MAB487) or an IgG2B isotype control (40 ng/mL; R&D Systems, MAB0061); (2) NIH‐3T3 cells were pretreated for 6 h with the CCR1 inhibitor BX471 (20 µM) or DMSO vehicle control (0.05%) prior to co‐culture.

To investigate the role of the STAT3 pathway in S100A8/A9‐mediated CCL6 production, BMDMs were stimulated with recombinant S100A8‐S100A9 heterodimer (1 µg/mL; HY‐P71076; MCE, USA) for 2 h. To identify the functional receptors mediating S100A8/A9‐induced STAT3 activation, BMDMs were pretreated with the TLR4 inhibitor TAK‐242 (1 µM; HY‐11109; MCE, USA) or the RAGE inhibitor FPS‐ZM1 (1 µM; HY‐19370; MCE, USA) for 1 h. For STAT3 pathway blockade, BMDMs were pretreated with the STAT3 inhibitor Stattic (5 µM; HY‐13818; MCE, USA) for 1 h before stimulation. For rescue experiments, BMDMs were transfected with siRNA targeting S100a9, followed by treatment with or without the STAT3 activator Colivelin TFA (50 µg/mL; HY‐P1061A; MCE, USA), which was added to the culture medium 4 h prior to downstream analysis.

For treatment, HIFs were exposed to recombinant CCL15 (HY‐P7266, MCE, USA) at concentrations of 5, 10, or 20 ng/mL for 48 h; the 10 ng/mL dose was optimized for subsequent assays. Where indicated, HIFs were pretreated with the CCR1 inhibitor BX471 (20 µm; HY‐12080, MCE, USA) or DMSO vehicle control (0.05%) for 6 h.

### 5.9 Ultra‐Fast Quantitative Proteomics

BMDMs were transfected with siNC or si*S100a9* and subsequently cultured in serum‐free DMEM for 48 h; the culture supernatants were collected (*n* = 3 per group) for proteomic profiling, conducted by Tsingke Biotechnology (China). The integrated workflow encompassed protein extraction, enzymatic digestion, LC‐MS/MS, database searching, and comprehensive bioinformatic analysis.

### 5.10 Collagen Gel Contraction Assay

Rat tail collagen type I (#354236, Corning, USA) was neutralized and diluted to 1.4 mg/mL. NIH‐3T3 cells (2 × 10^5^ per 500 µL) were mixed with the collagen solution and plated in 24‐well plates. Following polymerization at 37°C for 20 min, cells were pretreated with BX471 (20 µm) or DMSO (0.05%) for 6 h prior to co‐culture with S100A8/A9^hi^ BMDMs. Gel diameters were measured at 0, 6, 12, and 24 h using ImageJ software (USA).

### 5.11 Fluorescent Enzyme Gene Reporter

The wild‐type (WT) and mutant *Ccl6* promoters were cloned into the pGL1 luciferase reporter vector. For the reporter assays, HEK‐293T cells were seeded in 24‐well plates and co‐transfected with a *Stat3*‐overexpressing plasmid (or an empty vector control) and the respective firefly luciferase reporter plasmid (WT or mutant) using Lipo8000 Transfection Reagent (C0533, Beyotime, China). All plasmids were synthesized by Corues Biotechnology (China). After 48 h, firefly and Renilla luciferase activities were measured sequentially using a Multi‐mode Microplate Reader (BioTek, USA) and the Dual Luciferase Reporter Gene Assay Kit (#11402ES60, YEASEN, China) according to the manufacturer's instructions.

### 5.12 Chromatin Immunoprecipitation (ChIP) Assay

ChIP assays were performed using the BeyoChIP Enzymatic ChIP Assay Kit A/G Kit (Protein A/G beads) (P2083S, Beyotime, China) according to the manufacturer's instructions. Briefly, BMDMs were stimulated with or without recombinant S100A8‐S100A9 heterodimer (1 µg/mL, HY‐P71076, MCE, USA) for 2 h to activate STAT3 signaling. Subsequently, the samples (4 × 10^6^ cells per condition) were cross‐linked with 1% formaldehyde for 10 min at 37°C, followed by quenching with 125 mm glycine. After cell lysis, chromatin was sheared via sonication to generate DNA fragments ranging from 150 to 1000 bp. A 2% aliquot of the sheared chromatin was reserved as the input control. The remaining chromatin was immunoprecipitated overnight at 4°C with 5 µg of anti‐pTyr705‐STAT3 antibody (# 9145, CST, UK) or an equal amount of species‐matched IgG control (# 3900, CST, UK). Following cross‐link reversal at 65°C for 6 h, the precipitated DNA was purified and eluted. The enrichment of p‐STAT3 binding at the *Ccl6* promoter was quantified by RT‐qPCR using the following primers: F: 5′‐GGGGACGAGTCTCAAAGCAA‐3′; R: 5′‐CTGTGGGAGGGCTTTCTGAG‐3′. The specificity of PCR amplification was further validated via agarose gel electrophoresis.

### 5.13 Figure Preparation

The graphic abstract was created with BioGDP.com [62].

### 5.14 Statistical Analysis

Statistical analyses were conducted using GraphPad Prism software (version 8.0; GraphPad Software, USA). All data are presented as the mean ± standard deviation (SD). Two‐group comparisons were made using unpaired, two‐tailed Student's t‐tests, while one‐way ANOVA was employed for comparisons involving three or more groups. A *p*‐value < 0.05 was considered statistically significant.

### 5.15 Ethics Approval and Consent to Participate

The clinical study was approved by the Ethics Committee of Nanjing Medical University [2025‐SR‐572]. All animal experimental procedures were approved by the Experimental Animal Care and Ethics Committee of Nanjing Medical University [IACUC‐2502066].

## Author Contributions

S.W. and J.W. contributed equally to this work and are co‐first authors. S.W., X.Z., and H.Z. conceptualized and designed the study. J.W. and Z.Y. conducted single‐cell data analysis. S.W., J.S., and J.L. performed the experiments and collected data. Y.Z., G.Z., and J.Y. carried out statistical analysis. C.J., N.T., and J.M. helped obtain clinical samples. S.W. wrote the original draft, and H.Z. and X.Z. revised it critically.

## Funding

This work was supported by grants from the National Natural Science Foundation of China (Grant Nos.82370535 and 82570616) and the China Postdoctoral Science Foundation (Grant Nos.2024M761212).

## Conflicts of Interest

The authors declare no conflicts of interest.

## Supporting information




**Supporting File**: advs76353‐sup‐0001‐SuppMat.docx.

## Data Availability

The mass spectrometry proteomics data have been deposited to the ProteomeXchange Consortiumvia the iProX partner repository with the dataset identifier PXD072481. (URL: https://www.iprox.cn/page/PSV023.html). All other data supporting this study's findings are available from the corresponding author upon reasonable request.
