# Supplementary material for: S100A8/A9‐High Macrophages Activate Intestinal Fibroblasts via mCCL6/hCCL15‐CCR1 Axis to Drive Intestinal Fibrosis in Crohn's Disease
Source: Adv Sci (Weinh). 2026 Jun 29;13(53):e76353. doi: 10.1002/advs.76353 (PMC13336410; doi:10.1002/advs.76353)
Supplement: Supplementary file 1 [file ADVS-13-e76353-s001.docx]

**S100A8/A9-High Macrophages Activate Intestinal Fibroblasts via mCCL6/hCCL15-CCR1 Axis to Drive Intestinal Fibrosis in Crohn's Disease**

*Shu Wang*^1^, *Jiayun Wang*^1^, *Junjie Lin*^1^, *Ziping Ye*^1^, *Geyujia Zhou*^1^, *Jingjing Ma*^1^, *Junjian Sun*^1^, *Jiang Yu*^1^, *Yingdi Zhang*^1^, *Nana Tang*^1^, *Chunhua Jiao*^1^, *Xiaojing Zhao*^1,*^, *Hongjie Zhang*^1,*^

^1^Department of Gastroenterology, The First Affiliated Hospital with Nanjing Medical University, 300 Guangzhou Road, Nanjing 210029, Jiangsu Province, China. ^*^e-mail: [hjzhang06@163.com (H](mailto:hjzhang06@163.com%20(H). Zhang). [zhaoxj91718@163.com (X](mailto:zhaoxj91718@163.com%20(X). Zhao).

**Supplemental Methods**

**Histological Staining and Assessment**

Intestinal tissue was fixed in 4% paraformaldehyde, paraffin-embedded, and sectioned at 4 μm. For general morphology, dewaxed sections were stained with hematoxylin for 5 min, differentiated in acid ethanol, and counterstained with eosin.^[1]^ For collagen assessment, adjacent sections were stained with Weigert's iron hematoxylin for nuclei, Biebrich scarlet-acid fuchsin for cytoplasm, and aniline blue for collagen fibers following phosphomolybdic acid differentiation.^[2]^

For semi-quantitative assessment, each specimen was divided into three distinct zones under low-power magnification: non-involved mucosa, actively inflamed area, and stricture segment. In each zone, 3 random high-power fields were evaluated for each patient. We adopted a semi-quantitative grading approach (0–3) for both inflammation and fibrosis assessment as previously described for stricture CD tissues.^[3 , 4]^ H&E-stained sections were scored for inflammation on a 4-grade scale of 0–3 (0 = no inflammation; 1 = mild, mucosal; 2 = moderate, mucosal/submucosal; 3 = severe, transmural). Masson's trichrome-stained adjacent sections were scored for fibrosis on a similar scale of 0–3 (0 = none; 1 = submucosal <25%; 2 = extending to muscularis propria; 3 = transmural), following the Modified Chiorean Score protocol. All assessments were conducted independently by two pathologists in a blinded fashion, with surgical resection margins serving as internal controls.

**Immunohistochemical (IHC) staining and quantification**

Paraffin-embedded intestinal tissue sections (4 μm) were dewaxed, rehydrated, and subjected to heat-induced antigen retrieval in citrate buffer. Endogenous peroxidase activity was quenched with 3% H₂O₂, followed by blocking with normal serum. Sections were incubated overnight at 4 °C with primary antibodies against S100A8/A9 (ab288715, Abcam, UK), CCL15 (OM273754, Omnimabs, USA), or CCL23 (DF2239, Affinity, USA), then with HRP-conjugated secondary antibody for 30 min at room temperature. Immunoreactivity was detected using DAB, and nuclei were counterstained with hematoxylin. Expression levels of S100A8/A9, CCL15 and CCL23 were quantified as integrated optical density (IOD) using ImageJ software on IHC-stained sections. Data were normalized to the total tissue area and presented as relative IOD.

**Western Blot** **Analysis**

Total, nuclear, and cytoplasmic proteins were extracted from cells. Specifically, nuclear and cytoplasmic fractions were isolated using the ExKine™ Protein Extraction Kit (KTP300, Abbkine, China) according to the manufacturer's instructions. Protein samples were separated by SDS-PAGE, transferred onto PVDF membranes, and incubated with the indicated antibodies as described previously.^[5]^ Protein bands were visualized using an enhanced chemiluminescence (ECL) system and quantified with ImageJ software (USA). When necessary, membranes were stripped with stripping buffer (Thermo, USA) for re-probing with additional antibodies. The antibodies used in our study were: anti-S100A8+S100A9 (ab288715, Abcam, UK), anti-Collagen I (ab138492, Abcam, UK), anti-Collagen VI (ab151422, Abcam, UK), anti-CCL6 (ab191400, Abcam, UK), anti-α-SMA (sc-53142, Santa Cruz, USA), anti-STAT3 Rabbit mAb (#12640, CST, USA) and phospho-STAT3-Y705 Rabbit mAb (#9145, CST, USA), CCR1 Rabbit mAb (A18341, ABclonal, China), anti-Lamin B1 (YA5343, MCE, USA) , anti-GAPDH (60004-1-Ig, Proteintech, China), HRP- conjugated, Goat Anti-Mouse IgG (H+L) (115-035-003, Jackson ImmunoResearch, USA) and Goat Anti-Rabbit IgG (H+L) (111-035-003, Jackson ImmunoResearch, USA) antibodies.

**Immunofluorescence Staining**

Following fixation, permeabilization, and blocking, cells or tissue sections were incubated with specific primary antibodies overnight at 4°C. Subsequently, the samples were incubated with appropriate fluorescently conjugated secondary antibodies at room temperature for 1 h in the dark. For cell proliferation analysis, the BeyoClick™ EdU Cell Proliferation Kit with AF594 (#C0078S, Beyotime, China) was performed according to the manufacturer's protocol. Fluorescence images were acquired using a Thunder Imager microscope (Leica DMi8, Germany). Antibodies used were: anti-CD68 (66231-2-Ig, Proteintech, China), anti-α-SMA (sc-53142, Santa Cruz, USA), anti-S100A8+S100A9 (ab288715, Abcam, UK), anti-F4/80 (17-4801-82, eBioscience, USA), anti-Collagen I (ab138492, Abcam, UK), anti-Fibronectin (ab46688, Abcam, UK), anti-Ki67 (ab16667, Abcam, UK), anti-phospho-STAT3-Y705 Rabbit mAb (#9145, CST, USA), anti-CCR1 Rabbit mAb (A18341, ABclonal, China), anti-CCL15 (PK55043, Abmart, China), anti-TLR4 (T62186, Abmart, China) and DAPI (BL 105A, Biosharp, China). Multiplex immunofluorescence staining was performed using the TSA Plus Kit (#G1256, Servicebio, China) according to the manufacturer's protocol. Briefly, tissue sections were sequentially incubated with antibodies against S100A8/A9, CD68, CCL15, CCR1, and α-SMA, each followed by tyramide signal amplification (TSA) with the corresponding Opal fluorophore. Slides were scanned using a digital slide scanner (Pannoramic MIDI, 3DHISTECH, Hungary) and analyzed with SlideViewer software (version 2.9.0.229983, 3DHISTECH, Hungary).

**Reverse transcription quantitative real-time PCR (RT-qPCR)**

Total RNA was extracted using TRIzol reagent (Invitrogen, USA) and subsequently reverse-transcribed into cDNA using a PrimeScript RT reagent kit (Vazyme Biotech, China). Subsequently, quantitative real-time PCR (qPCR) was performed using SYBR Green Master Mix (Vazyme Biotech, China) on an ABI 7300 Real-Time PCR System (Thermo Scientific, USA). All primer sequences were synthesized by Tsingke Biotechnology (China) and are listed in Supplementary Table 2.

**Isolation and Culture of Bone Marrow-Derived Macrophages (BMDMs)**

BMDMs were harvested from the tibias and femurs of 6-week-old wild-type (WT) C57BL/6 mice as previously described.^[6]^ Briefly, the bone marrow cells were flushed and cultured in Dulbecco's modified Eagle's medium (DMEM, Gibco, USA) supplemented with 10% FBS (AB-FBS-1050S, ABW, Uruguay) and 1% penicillin-streptomycin (NCM Biotech, China). To induce macrophage maturation, recombinant M-CSF (10 ng/mL, #P07141, GenScript, China) was added to the culture medium. The medium was refreshed every two days, and the cells were fully differentiated and ready for subsequent experiments by day 7.

**Histological score**

Histopathological evaluation of hematoxylin and eosin **(**H&E)-stained colon sections of colitic mice employed a semi-quantitative system that incorporated scores for epithelial damage (0-4) and inflammatory cell infiltration (0-4).^[7]^

**Isolation of murine lamina propria mononuclear cells**

Murine lamina propria mononuclear cells (LPMCs) were isolated from colonic tissues as previously described.^[8]^ Briefly, to remove the epithelial layer, colon tissues were incubated in EDTA-PBS buffer (2% FBS, 0.5 mM EDTA, 10 mM HEPES). The remaining fraction was then digested in RPMI 1640 medium containing 1% penicillin-streptomycin, 20% FBS, and 100 U/mL collagenase I (#1904GR001, Biofroxx, Germany). After passing the digest through a 70-μm cell strainer, the liberated cells were collected, resuspended in 40% Percoll-PBS. This suspension was carefully layered over 75% Percoll (#17089102, Cytiva, USA). After gradient centrifugation, LPMCs were harvested from the interface for subsequent flow cytometry analysis.

**Flow cytometry**

To evaluate the impact of S100A8/A9^hi^ BMDMs on fibroblast proliferation, co-cultured NIH-3T3 fibroblasts were analyzed using an EdU assay kit (Beyotime, China). The efficiency of macrophage depletion in mice was validated by staining splenic single-cell suspensions with APC-conjugated anti-F4/80 (17-4801-82, eBioscience, USA) and PE-conjugated anti-CD11b (12-0112-82, eBioscience, USA) antibodies. To identify S100A8/A9-positive macrophages among LPS-stimulated BMDMs or colonic LPMCs from Paquinimod (PAQ)-treated colitic mice, cells were first surface-stained with APC APC-conjugated anti-F4/80 and PE-conjugated anti-CD11b. Following fixation and permeabilization with the Fix & Perm Kit (GAS005, Multi Sciences, China), cells were incubated with anti-S100A8/A9 primary antibody (ab288715, Abcam, UK) for 1 h, followed by a FITC-conjugated secondary antibody (ab150077, Abcam, UK) for 1 h. To investigate whether mouse CCL6 (mCCL6) derived from S100A8/A9^hi^ BMDMs promotes fibroblast proliferation, co-cultures were treated with either a CCL6-neutralizing antibody or the CCR1 inhibitor BX471. Subsequently, fibroblast proliferation was assessed via Ki67 immunostaining (ab16667, Abcam, UK) or EdU assay (Beyotime, China). All flow cytometry data were acquired on a CytoFLEX S cytometer (Beckman Coulter) and analyzed using FlowJo software (BD, USA).

**Enzyme-linked immunosorbent assay (ELISA)**

Concentrations of murine CCL6 in BMDM culture supernatant and in colon tissues and serum from experimental mice, as well as human CCL15 (hCCL15) levels in serum from CD patients with or without intestinal fibrosis, were quantified using commercially available ELISA kits (Shanghai Hengyuan Biological Technology Co., Ltd., China) according to the manufacturer's instructions.

**Wound healing scratch assay**

Cells were seeded into 6-well plates at 2 × 10⁶ cells/well and cultured until confluent. A straight scratch was made across the monolayer using a sterile 200-μL pipette tip. After washing three times with PBS, cells were incubated with serum-free medium containing the indicated treatments. Wound closure was photographed at 0 and 24 h under a phase-contrast microscope (Olympus, Japan), and the denuded area was quantified using ImageJ.

**Supplementary Reference**

1. I. C. Allen, J. E. Wilson, M. Schneider, et al., "Nlrp12 Suppresses Colon Inflammation and Tumorigenesis through the Negative Regulation of Noncanonical Nf-Κb Signaling," *Immunity* 36, no. 5 (2012): 742-754. <https://doi.org/10.1016/j.immuni.2012.03.012>

2. M. Chvapil, D. P. Speer, J. A. Owen, T. A. Chvapil, and M. Chvapil, "Identification of the Depth of Burn Injury by Collagen Stainability," *Plastic and Reconstructive Surgery* 73, no. 3 (1984): 438-441. <https://doi.org/10.1097/00006534-198403000-00018>

3. I. O. Gordon, D. Bettenworth, A. Bokemeyer, et al., "Histopathology Scoring Systems of Stenosis Associated with Small Bowel Crohn's Disease: A Systematic Review," *Gastroenterology* 158, no. 1 (2020): 137-150.e131. <https://doi.org/10.1053/j.gastro.2019.08.033>

4. A. Coimbra, J. Rimola, M. Cuatrecasas, et al., "Magnetic Resonance Enterography and Histology in Patients with Fibrostenotic Crohn's Disease: A Multicenter Study," *Clinical and Translational Gastroenterology* 13, no. 7 (2022): e00505. <https://doi.org/ARTN> e00505

10.14309/ctg.0000000000000505

5. P.-C. Yang, and T. Mahmood, "Western Blot: Technique, Theory, and Trouble Shooting," *North American Journal of Medical Sciences* 4, no. 9 (2012): 429-434. <https://doi.org/10.4103/1947-2714.100998>

6. M. K. Warren, and S. N. Vogel, "Bone Marrow-Derived Macrophages: Development and Regulation of Differentiation Markers by Colony-Stimulating Factor and Interferons," *Journal of Immunology* 134, no. 2 (1985): 982-989.

7. A. H. Fischer, K. A. Jacobson, J. Rose, and R. Zeller, "Hematoxylin and Eosin Staining of Tissue and Cell Sections," *Cold Spring Harbor Protocols* 2008, no. 5 (2008): pdb.prot4986. <https://doi.org/10.1101/pdb.prot4986>

8. S. Wang, Y. Xu, L. Wang, et al., "Toldc Restores the Balance of Th17/Treg Via Aryl Hydrocarbon Receptor to Attenuate Colitis," *Inflammatory Bowel Diseases* 30, no. 9 (2024): 1546-1555. <https://doi.org/10.1093/ibd/izae022>

**Supplemental Table 1: Clinical characteristics of study participants.**

|  | CD patients without intestinal fibrosis | CD patients with intestinal fibrosis |
| --- | --- | --- |
| Number | 20 | 20 |
| Age (years) | 33.40±14.11 | 39.65±13.12 |
| Gender |  |  |
| Male | 14 (70%) | 12 (60%) |
| Female | 6 (30%) | 8 (40%) |
| Course of disease (months) | 39.25±13.69 | 59.20±37.41^a)^ |
| Disease location  L1  L2  L3  L4 | 14(70%)  0 (0%)  5(25%)  1(5%) | 7 (35%)  0 (0%)  11 (55%)  2 (10%) |
| CRP (mg/L) | 7.91±6.21 | 11.21±8.62 |
| ESR (mm/h) | 17.05±10.81 | 25.05±16.46 |
| WBC (10^^^9/L) | 6.55±1.42 | 6.25±2.11 |
| Fecal calprotectin (μg/g) | 441.57±310.29 | 495.94±273.06 |

All values are expressed as mean ± SD, a) *p* < 0.05.

**Supplemental Table 2: Primer sequences for gene expression analysis.**

| Gene | Forward sequence (5’-3’) | Reverse sequence (5’-3’) |
| --- | --- | --- |
| *Il-1b* (mouse) | GAAATGCCACCTTTTGACAGTG | TGGATGCTCTCATCAGGACAG |
| *Il-6* (mouse) | CTGCAAGAGACTTCCATCCAG | AGTGGTATAGACAGGTCTGTTGG |
| *Tnf-α* (mouse) | CTGAACTTCGGGGTGATCGGC | GGCTTGTCACTCGAATTTTGAGA |
| *Ifn-g* (mouse) | TCGGTAACTGACTTGAATGTCCA | TCGCTTCCCTGTTTTAGCTGC |
| *S100a8* (mouse) | AAATCACCATGCCCTCTACAAG | CCCACTTTTATCACCATCGCAA |
| *S100a9* (mouse) | GCACAGTTGGCAACCTTTATG | TGATTGTCCTGGTTTGTGTCC |
| *Ccl6* (mouse) | AAGAAGATCGTCGCTATAACCCT | GCTTAGGCACCTCTGAACTCTC |
| *Ccr1* (mouse) | ACTGCTGTAAGAGCCTTTGGG | AGCACCAGAATCACTAGGACA |
| *Col1a1* (mouse) | GCTCCTCTTAGGGGCCACT | ATTGGGGACCCTTAGGCCAT |
| *Col6a1* (mouse) | CTGCTGCTACAAGCCTGCT | GCACGAAGAATAGATCCACAGGG |
| *Col3a1* (mouse) | CTGTAACATGGAAACTGGGGAAA | CCATAGCTGAACTGAAAACCACC |
| *Fn1* (mouse) | ATGTGGACCCCTCCTGATAGT | GCCCAGTGATTTCAGCAAAGG |
| *Acta2* (mouse) | CCCAGACATCAGGGAGTAATGG | TCTATCGGATACTTCAGCGTCA |
| *Gapdh* (mouse) | AGGTCGGTGTGAACGGATTTG | GGGGTCGTTGATGGCAACA |
| *ACTA2* (human) | AAAAGACAGCTACGTGGGTGA | GCCATGTTCTATCGGGTACTTC |
| *COL1A1* (human) | GAGGGCCAAGACGAAGACATC | CAGATCACGTCATCGCACAAC |
| *COL6A1* (human) | ACAGTGACGAGGTGGAGATCA | GATAGCGCAGTCGGTGTAGG |
| *COL6A3* (human) | ATGAGGAAACATCGGCACTTG | GGGCATGAGTTGTAGGAAAGC |
| *FN1* (human) | CGGTGGCTGTCAGTCAAAG | AAACCTCGGCTTCCTCCATAA |
| *COL3A1* (human) | GGAGCTGGCTACTTCTCGC | GGGAACATCCTCCTTCAACAG |
| *CCL15* (human) | TCCCAGGCCCAGTTCATAAAT | TGCTTTGTGAGATGTAGGAGGT |
| *CCR1* (human) | GACTATGACACGACCACAGAGT | CCAACCAGGCCAATGACAAATA |
| *CCR3* (human) | TGGCATGTGTAAGCTCCTCTC | CCTGTCGATTGTCAGCAGGATTA |
| *GAPDH* (human) | GGAGCGAGATCCCTCCAAAAT | GGCTGTTGTCATACTTCTCATGG |

**Supplemental Table 3: List of siRNAs for *S100a9.***

| Gene | Forward sequence (5’-3’) | Reverse sequence (5’-3’) |
| --- | --- | --- |
| si*S100a9*-1 | CCAAGAAGGAAUUCAGACA | UGUCUGAAUUCCUUCUUGC |
| si*S100a9*-2 | CAGAUGGAGCGCAGCAUAA | UUAUGCUGCGCUCCAUCUG |
| si*S100a9*-3 | GACAAAUGGUGGAAGCACA | UGUGCUUCCACCAUUUGUC |
| *Mus-Negative Control* | UUCUCCGACAGUGUCACGU | ACGUGACACUGUCGGAGAA |

**Supplemental Table 4: Complete blood count and liver/kidney function of Paquinimod-treated colitic mice.**

|  | Control | DSS+Vehicle | DSS+PAQ (1mg/kg/d) | DSS+PAQ (5mg/kg/d) | DSS+PAQ (10mg/kg/d) | Reference range |
| --- | --- | --- | --- | --- | --- | --- |
| WBC (*10^9^/L) | 5.38±1.654 | 3.86±1.212 | 3.38±0.327 | 3.3±0.570 | 4.84±0.541 | 0.8-10.6 |
| Lymph (*10^9^/L) | 3.08±1.150 | 2.78±0.947 | 2.74±0.260 | 2.86±0.643 | 3.58±0.383 | 0.6-8.9 |
| Mon (*10^9^/L) | 0.44±0.241 | 0.66±0.288 | 0.24±0.114 | 0.12±0.084 | 0.16±0.089^d)^ | 0.04-1.4 |
| Gran (*10^9^/L) | 1.08±0.492 | 1.26±0.488 | 0.68±0.130 | 0.5±0.158 | 0.76±0.230 | 0.23-3.6 |
| Lymph (%) | 68.52±7.11 | 64.04±7.655 | 73.38±3.357 | 81.7±6.077 | 72.94±2.512^c)^ | 40-92 |
| Mon (%) | 3.96±0.976 | 4.42±0.554 | 5.44±0.623 | 3.48±0.563 | 3.42±0.415^c)^ | 0.9-18 |
| Gran (%) | 21.12±3.531 | 28.018±3.183 | 24.22±5.069 | 15.36±2.479 | 22.52±1.825^c)^ | 6.5-50 |
| RBC (*10^12^/L) | 11.148±0.631 | 10.66±0.727 | 9.992±1.141 | 9.948±1.122 | 9.864±1.026 | 6.5-11.5 |
| HGB (g/L) | 157.2±4.550 | 146.6±8.414^a)^ | 133.4±11.149 | 129.8±12.518 | 142±14.018 | 110-165 |
| PLT (*10^9^/L) | 1018.6±126.599 | 1332.2±57.838^b)^ | 1429.6±61.962 | 1399.6±171.188 | 1299±232.532 | 400-1600 |
| ALT(U/L) | 25.0602±8.075 | 20.4656±4.236 | 30.5764±4.680 | 26.821±4.349 | 28.932±4.429^c)^ | 10.06-96.47 |
| AST (U/L) | 115.383±13.985 | 97.8992±15.972 | 114.9208±8.173 | 124.047±37.265 | 101.9248±9.133 | 36.31-235.48 |
| CREA (μmol/L) | 15.2104±1.037 | 15.6898±1.643 | 15.4456±1.145 | 14.1062±0.94 | 14.0066±1.012 | 10.91-85.09 |

a) *p* < 0.05, b) *p* < 0.01 compared with the Control group; c) *p* < 0.05, d) *p* < 0.01 compared with the DSS+Vehicle group. Abbreviations**:** WBC, white blood cell; lymphocyte; Mon, monocyte; Gran, granulocyte; RBC, red blood cell; HGB, hemoglobin; PLT, platelet; ALT, alanine aminotransferase; AST, aspartate aminotransferase; CREA, creatinine. All values are expressed as mean ± SD.

**Supplemental Figures**

**Figure S1. Identification and characterization of myeloid cell subsets in Crohn’s disease (CD) intestinal lesions.** A single-cell RNA sequencing dataset of full-thickness intestinal specimens from CD patients, comprising non-involved, inflamed, and stricture regions, was obtained from Prof. Florian Rieder and reanalyzed. A) Dot plot showing gene expression signatures defining distinct myeloid cell subpopulations. B) Dot plots of representative markers across major cell lineages. In both panels, dot color and size represent the mean expression level and the percentage of cells expressing the gene, respectively.

**Figure S2. Induction of S100A8/A9^hi^ BMDMs.** A) Bone marrow-derived macrophages (BMDMs) were stimulated with LPS (100 ng/mL), IL-1β (100 ng/mL), or TNF-α (100 ng/mL) for 6, 12, and 24 h. mRNA expression levels of *S100a8* and *S100a9* were quantified by RT-qPCR. B) BMDMs were treated with LPS (100 ng/mL) for 24 h; the proportion of S100A8/A9^+^ cells was determined by flow cytometry (FCM). C) Colitic mice received an intravenous injection of clodronate liposomes (200 μL/mouse). The depletion efficiency of macrophages in splenic single-cell suspensions was assessed by FCM. All values are expressed as mean ± SD. ns, not significant, ^**^*p* < 0.01, ^***^*p* < 0.001.

**Figure S3. Successful recruitment of adoptively transferred macrophages to the colons of mice with colitis.** BMDMs transfected with control siRNA (siNC) or si*S100a9* were labeled with the lipophilic fluorescent dye DiR and adoptively transferred into mice with DSS-induced colitis. A) The homing and persistence of the transferred cells were monitored longitudinally using an In Vivo Imaging System (IVIS) at the indicated time points (1, 6, 24, and 48 h). B, C) At the experimental endpoint, colon tissues and major organs (lung, heart, spleen, liver, and kidney) were harvested for *ex vivo* fluorescence imaging and quantification using IVIS. D) Representative fluorescence microscopy images showing the infiltration of DiR-labeled cells in colonic tissue sections. Scale bar: 100 μm. All values are expressed as mean ± SD. ^*^*p* < 0.05, ^**^*p* < 0.01, ^***^*p* < 0.001.

**Figure S4. Paquinimod (PAQ) alleviates intestinal inflammation in mice with DSS-induced** **chronic colitis.** WT mice were subjected to three cycles of 2% DSS and were administered PAQ (1, 5, or 10 mg/kg/day) via oral gavage. A) Body weight was monitored every two days, and the percentage of body weight was calculated. B) Representative macroscopic images of colons and total colon length measurements. C) mRNA expression levels of pro-inflammatory cytokines (*Il-1b*, *Tnf-α*, *S100a8*, *S100a9*, and *Ccl6*) in colonic tissues were determined by RT-qPCR. All values are expressed as mean ± SD. ^*^*p* < 0.05, ^**^*p* < 0.01, ^***^*p* < 0.001.

**Figure S5. S100A8/A9^hi^ macrophage-derived mCCL6 exacerbates intestinal inflammation and fibrosis in chronic colitis.** Mice with DSS-induced colitis were adoptively transferred with either NC-BMDMs or S100A8/A9^hi^ BMDMs. Beginning at the first DSS cycle, mice in the S100A8/A9^hi^ BMDM group were administered either a CCL6-neutralizing antibody or an isotype control (4 µg/mouse, intraperitoneally) every three days. A) Bar plot showing differentially expressed proteins. B, C) CCL6 concentrations in colonic tissues(B) and serum(C) were determined by ELISA. D) Changes in body weight over the experimental period. E) Representative macroscopic appearance and statistical analysis of colon length. F) Transcriptional levels of inflammatory cytokines (*Il-1b, Il-6, Tnf-α*) were quantified by RT-qPCR. G) mRNA expression levels of fibrosis-associated genes (*Col1a1*, *Col6a1*, *Col6a3*, *Col3a1* and *Fn1*) were measured via RT-qPCR. All values are expressed as mean ± SD. ns, not significant, ^*^*p* < 0.05, ^**^*p* < 0.01, ^***^*p* < 0.001.

**Figure S6. S100A8/A9^hi^ macrophages promote fibroblast** **proliferation, activation, and extracellular matrix production via CCL6.** NIH-3T3 fibroblasts were co-cultured with S100A8/A9^hi^ BMDMs at a 1:2 ratio for 48 h. For CCL6 neutralization, S100A8/A9^hi^ BMDMs were pretreated with either a CCL6 neutralizing antibody or an IgG2B isotype control (40 ng/mL each) for 6 h prior to co-culturing with fibroblasts. A) Fibroblast proliferation was assessed by EdU incorporation assay. Scale bar: 100 µm. B) Proliferating fibroblasts were stained for Ki67 and quantified by flow cytometry. C) Fibroblast activation was evaluated by α-SMA immunofluorescence. Scale bar, 100 µm. D) Protein expression levels of COL1A1, COL6A1, and α-SMA in NIH-3T3 cells were determined by Western blot. All values are expressed as mean ± SD. ns, not significant, ^*^*p* < 0.05, ^**^*p* < 0.01, ^***^*p* < 0.001.

**Figure S7. S100A8/A9^hi^ macrophage-derived CCL6 promotes fibroblast activation, ECM production, migration, and collagen contraction via CCR1.** Following 48-h co-culture with S100A8/A9^hi^ BMDMs at a 1:2 ratio, NIH-3T3 fibroblasts were analyzed for CCR1 expression. A-C) The expression of CCR1 in NIH-3T3 cells was measured by RT-qPCR (A), Western blot (B) and immunofluorescence staining. Scale bar: 100 µm (C). For inhibitor experiments, S100A8/A9^hi^ BMDMs were pretreated with a CCR1 inhibitor (BX471, 20 µM) for 6 h before co-culture. D) Proliferating fibroblasts stained with EdU were quantified by flow cytometry. E) Western blot analysis of CCR1 and fibroblast ECM components (COL1A1, COL6A1, and α-SMA). F) Fibroblast migration was evaluated using a wound healing assay and quantified by measuring the wound closure area. Scale bar: 500 µm. G) Fibroblast contractility was assessed by collagen gel contraction assay and quantified by measuring gel diameter at 0, 6, 12, and 24 h. Representative images at 0 and 24 h are shown (left). The data are presented as mean ± SD from three independent experiments. ns, not significant, ^*^*p* < 0.05, ^**^*p* < 0.01, ^***^*p* < 0.001.

**Figure S8.** **Human CCL15 (hCCL15) drives fibroblast proliferation, activation, migration, and collagen production via CCR1.** A) Expression levels of CCL15 and CCL23 in human tissues and organs based on data from the Human Protein Atlas. B) mRNA levels of *CCL23* in paired non-stricture and stricture intestinal regions from CD patients. C) Representative immunohistochemical staining images and quantification of CCL23 in non-stricture and stricture surgical tissue sections from CD patients. Scale bar: 500 µm (low magnification), scale bar: 100 µm (high magnification). n = 4 patients; 3 fields per zone. D) *CCR1* and *CCR3* expression levels in fibroblast subclusters were visualized in scRNA-seq data. E, F) RT-qPCR analysis of *CCR1* and *CCR3* expression in paired non-stenotic and stenotic colonic tissues from patients with CD (E), and in primary fibroblasts isolated from these regions (F). Human intestinal fibroblasts were treated with recombinant CCL15 (10 ng/mL, 48 h) with or without pretreatment with the CCR1 inhibitor (BX471,20 µM). G) Protein levels of CCR1 and COL1A1 were detected by Western blot. H) Quantitative analysis of fibroblast migration rates. Scale bars: 500 µm. I) Representative immunofluorescence images of Ki67 (proliferation marker) and α-SMA (activation marker) in fibroblasts. Scale bar: 100 µm. Data are expressed as mean ± SD from three independent experiments. ns, not significant, ^*^*p* < 0.05, ^**^*p* < 0.01, ^***^*p* < 0.001.
